# An evaluation scale for the cultural value of heritage buildings

**DOI:** 10.1371/journal.pone.0350924

**Published:** 2026-06-23

**Authors:** Hai Huang, Mohd Khairul Azhar Mat Sulaiman, Nor Zalina Harun, Wanting He

**Affiliations:** 1 Department of Architecture and Built Environment, Faculty of Engineering and Built Environment, Universiti Kebangsaan Malaysia, Bangi, Malaysia; 2 The School of Art and Design Engineering, Chongqing Vocational Institute of Engineering, Jiangjin, Chongqing, China; 3 KPU Senibina dan Alam Bina Inovatif, Universiti Kebangsaan Malaysia, Bangi, Malaysia; 4 Institute of the Environment and Development, Universiti Kebangsaan Malaysia, Bangi, Malaysia; 5 School of Design, Chongqing Industry Polytechnic University, Yubei, Chongqing, China; Hunan University, CHINA

## Abstract

As a repository of human history and a conduit for culture, heritage buildings represent the primary central focus of heritage conservation. However, the lack of a comprehensive framework for evaluating the cultural value of heritage buildings is a significant challenge. Based on a systematic review of the classification of cultural value in cultural heritage over the past two decades, this study employed face-to-face interviews with ten local shop owners, go-along interviews with ten visitors, and a Focus Group Discussion (FGD) with five experts in Longxing Ancient Town, Chongqing, China. The interview transcripts were coded and analyzed with NVivo software, the FGD results were analyzed manually, and the weights of the indicators were assigned using the Analytical Hierarchy Process (AHP). Ultimately, by synthesising the perspectives of various stakeholders, the Cultural Value Evaluation Scale (CVES) for heritage buildings was developed for the first time. This scale, designated as the LHSSA Framework (Local value, Historic value, Sustainability value, Scientific value, Aesthetic value), encompasses five primary indicators and 24 secondary indicators. The scale offers considerable theoretical significance and practical value for the identification of heritage value and the improvement of the heritage value evaluation system.

## 1. Introduction

Heritage buildings play an integral role in the daily and spiritual lives of human beings, serving as a conduit for numerous human civilizations and invaluable memories [1]. Concurrently, heritage buildings are acknowledged as a valuable, distinctive, limited, and irreplaceable asset of a nation’s wealth and historical standing [2]; they are accorded the primary objective of conservation [3]. While the cultural presence in heritage buildings may leave tangible evidence, it is often overlooked because such evidence is frequently inaccessible or intangible [4]. UNESCO (2005) stated that “parties should endeavor to integrate culture into their development policies at all levels to create conditions conducive to sustainable development”. In the absence of an awareness of the cultural values of these spaces, practices, and histories, some anachronistic developments may undermine the unique spatial identity and cultural significance of the locality, ultimately leading to the rupture of the community and its history [5]. The “100 percent heritage” approach posited that heritage buildings should be conserved and advocated for a more nuanced definition of what constitutes heritage and the rationale for its preservation [6,7]. In this context, a concise framework for evaluating the cultural value of heritage buildings can be a useful tool for effectively informing conservation decisions and ensuring a comparative baseline for future value evaluations and conservation impact evaluations of heritage buildings.

Over the past two decades, the cultural value of heritage buildings has been elucidated from a multitude of perspectives. Heritage buildings constitute an integral aspect of contemporary society, encompassing notable edifices of historical and architectural significance [8]. In 1972, UNESCO published a framework for categorizing the cultural values of heritage, comprising historic value, aesthetic/artistic value, scientific value, and social value [9]. Subsequently, scholars have expanded this categorization framework, incorporating additional values such as educational [10], spiritual, symbolic [11], political, economic [12], and ecological values [13,14]. However, these categories offer little innovation and instead tend to confuse readers. For example, whether educational value intersects with historic value [10], whether symbolic and aesthetic values are duplicated [11], and why ecological and economic values are subordinate to cultural values [14]. Despite efforts by scholars to expand and refine the categories of cultural value, the current categorization framework does not accurately convey the true connotation of the cultural value of heritages. Besides, general heritage building evaluation tools, such as Building Research Establishment Environmental Assessment Method (BREEAM), make only limited reference to culture-related issues [15]. Heritage Impact Assessment (HIA) provides a structured procedure to evaluate the effects of development on the cultural values and attributes of heritage places, and UNESCO/ICOMOS guidance has formalized its use in World Heritage contexts [16]. These frameworks and tools may appear to offer valuable insight, yet they cannot evaluate the cultural value of heritage buildings.

The extant literature indicates that tools for evaluating the cultural value of heritage buildings are indispensable for decision-making at the implementation of heritage conservation objectives [17,18]. However, the Saudi Commission for Tourism and National Heritage (2017) indicated that 45% of the world’s heritage buildings are in a state of disrepair due to a lack of maintenance programs. The absence of a precise definition of heritage buildings has resulted in the erroneous demolition of some heritage buildings located in residential neighborhoods [19]. A framework for evaluating the cultural value of heritage buildings can assist in decision-making processes related to value evaluation, planning and design, and protection strategies in conservation projects. Scholars have explored the evaluation of heritage values in a multitude of ways [20,21]. Nevertheless, the extant methodologies for evaluating the cultural value of heritage buildings remain deficient in its integration of context-specific and cultural considerations [22]. For such a framework to be effective, it is necessary to have a concise set of indicators that adequately cover the core elements of the cultural value of heritage buildings. Such a set of indicators should cover the physical characteristics of heritage buildings, measure the cultural value of heritage buildings, define the limits of change for each description [23], provide a common language to be used amongst stakeholders [17], and evaluate the cultural value through people’s perception of the physical characteristics of the buildings. This paper addresses two key questions: (1) How can the criteria for categorizing the cultural value of heritage buildings be made as comprehensive as possible? (2) What are the perceptions of the cultural value of heritage buildings held by different stakeholders?

In these contexts, this study presented a theoretical analysis and empirical extension of existing evaluation frameworks to identify common indicators and priorities for evaluating the cultural value of heritage buildings. Based on this, a set of straightforward, accessible, and quantifiable criteria is developed to facilitate decision-making in the conservation process. The Cultural Value Evaluation Scale (CVES), developed based on these indicators, comprises five primary indicators and 24 secondary indicators, providing both a theoretical foundation and practical tools for the quantitative evaluation of heritage’s intangible cultural value for the first time, while offering crucial guidance for formulating precise conservation strategies in the preservation of heritage buildings.

## 2. Theoretical basis

Šćekić et al. (2025) identified 151 distinct types of cultural heritage value, with cultural value being repeatedly cited by various institutions [24]. The definition of cultural value consists of three elements. First, cultural value is an object that is perceived by humans. Second, the processes and outcomes of perception are abstract, yet these outcomes possess a multifaceted value. Third, cultural values exert an influence on human behavior and practices [25–31]. The foundation of heritage buildings protection is the accurate perception of their value, particularly in terms of cultural value. Some scholars have diverged from the categorization framework proposed by UNESCO, conducting new explorations. For instance, Stephenson (2005) developed a model of cultural value in the landscape, defining cultural value as forms, relationships, and practices [29]. Olukoya proposed a value evaluation framework-the Vernacular Value Model (VVM), and drew on previous research to incorporate a variety of typical types of “value markers”, including aesthetic/artistic value, historic value, cultural/symbolic value, social/community value, and economic value [28]. While these abstract frameworks offer innovative insights, they are not straightforward to comprehend, let alone serve as tools for evaluating cultural values. Through further analysis, most of the frameworks are still only substitutes for UNESCO’s classification framework. Therefore, it is necessary to re-identify and summarize these classification criteria.

**Fig 1:**
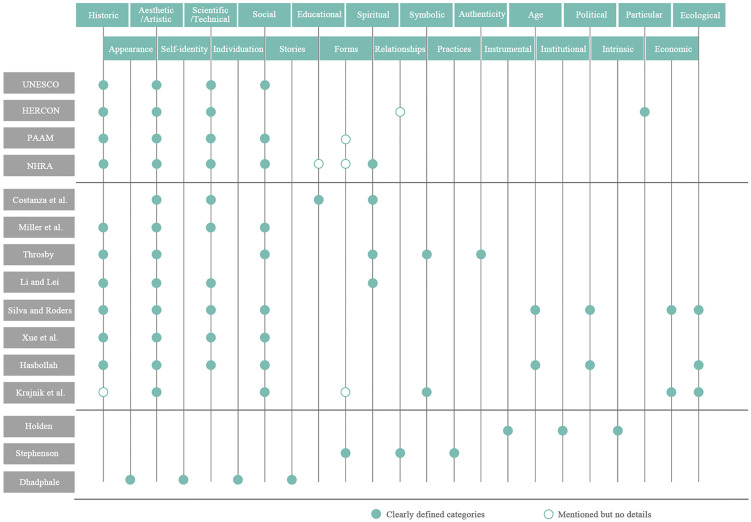
Classification of cultural values in the literature.

From a theoretical standpoint, researchers have conducted a systematic review of studies on the categorization of cultural value in cultural heritage from 2005 to 2025 [32], which constitutes the core theoretical foundation of the CVES. This review encompassed the categories of cultural values proposed by prominent organizations, the categories of cultural values expanded by scholars, and other abstract categorization frameworks (Fig 1). In addition, the systematic overview presented a novel perspective. Given the expansive nature of social value, Huang et al. (2025) excluded it from the UNESCO classification framework as the connotation of social value can be adequately captured by other values. Furthermore, the researcher subdivided some of the categories into subcategories that were encompassed by the main categories. Furthermore, the study introduced the concept of “local/place value” as a consequence of considering the perspectives of various stakeholders on heritage buildings. The researcher ultimately established a categorization framework of cultural values, comprising four main categories and 14 subcategories [32], as illustrated in Table 1.

**Table 1:** Categories of cultural values summarized in the systematic literature review.

Primary categories	Subcategories	Category description
Historic Value	Time-honored	Length of history.
	Symbolic	The object was involved in or associated with important events in the past.
	Educational	Heritage objects are where the potential for the future to understand the past lies.
	Conceptual	Integral materialization of conceptual intentions.
Local/ Place Value	Belong to the local	It exists within a certain geographical scope.
	Circulated in full	The way of transmission from one generation to another.
	Conceptual	Integral materialization of conceptual intentions.
Artistic/ Aesthetic Value	Artistic	Original product of creativity and imagination.
	Notable	Product of a creator, holding his signature.
	Evidential	Part of the history of art or architecture.
	Conceptual	Integral materialization of conceptual intentions.
Scientific/ Technical Value	Workmanship	Original result of human labor, craftsmanship.
	Technological	Skillfulness of techniques and materials, representing an outstanding quality of work.
	Conceptual	Integral materialization of conceptual intentions.

As evidenced by the categories of cultural value of heritage buildings derived from the systematic review, this framework appears to lack comprehensive coverage and is largely based on an expert perspective. Moreover, the framework is unable to adequately address the significance of cultural sustainability. Consequently, it is imperative to develop a CVES grounded in the classification of cultural values for heritage. This scale must encompass a broader scope and incorporate the perspectives of diverse stakeholders.

## 3. Methodology

### 3.1. Research design

**Fig 2:**
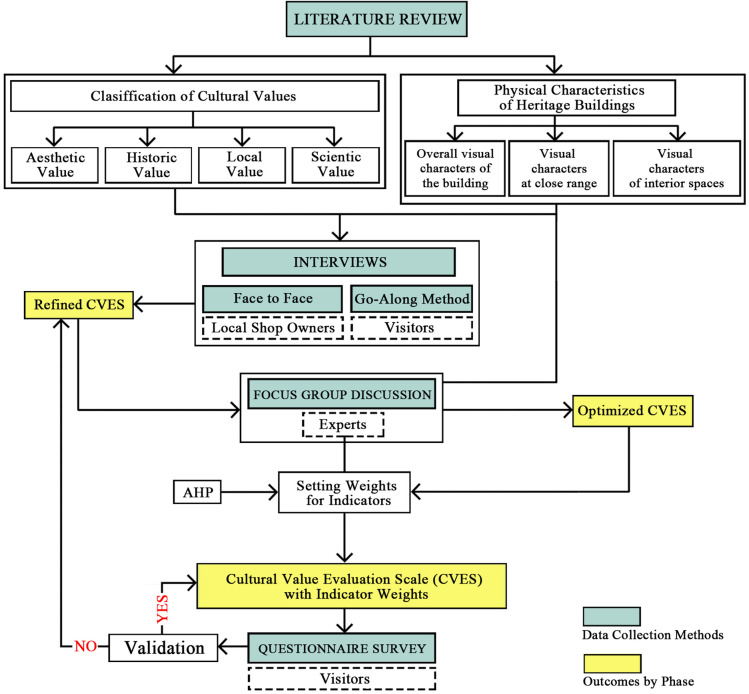
Diagram of the research design.

The cultural value of heritage was summarized into aesthetic/artistic value, historic value, local/place value, and scientific/technical value, as well as 14 subcategories through a systematic review, which served as the original basis for the CVES. Three major physical characteristics of heritage buildings were also summarized, namely overall visual characteristics, visual characteristics at close range, and internal spatial visual characteristics [33]. Subsequently, face-to-face interviews were conducted with local shop owners, and go-along interviews were conducted with visitors. The interview transcripts were coded and analyzed using NVivo software to refine the CVES. Finally, five experts were invited to a Focus Group Discussion (FGD) aimed at accomplishing two tasks: 1) to review, adapt, and add to the CVES; 2) to assign weights to the indicators in the scale. Then, this scale was used to conduct an online questionnaire survey among visitors to the ancient town, to validate its reliability and validity. If the validation results do not meet acceptable standards, the interview conclusions would be analyzed again until the validation results reached an acceptable level. Fig 2 illustrates the flow of the research design.

### 3.2. Area of study

**Fig 3:**
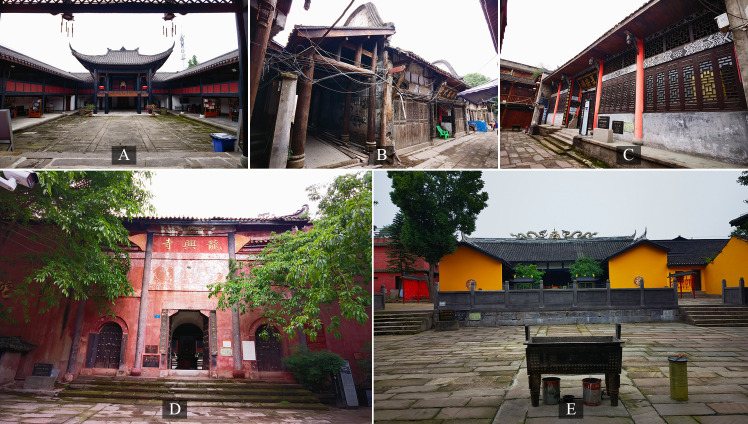
Heritage buildings in Longxing Ancient Town. **(A)** Liu Family Ancestral Hall, **(B)** Liu Family Courtyard, **(C)** Huaxia Ancestral Hall, **(D)** Longxing Temple, and **(E)** Longzang Palace.

Located in Liangjiang New District, Chongqing, China, Longxing Ancient Town has a history of more than 600 years, and is an important material carrier for the study of the development and evolution of regional culture. Since 2003, the Chinese Government has protected historical and cultural towns as a special category of heritage and has now gazetted 312 towns throughout the country, which are usually characterized by a long history and regional culture. Longxing Ancient Town is one of them. The most important heritage resources in Longxing Ancient Town are the existing heritage buildings, which are mainly religious or clan public buildings, among which five buildings, Liu Family Ancestral Hall, Liu Family Courtyard, Huaxia Ancestral Hall, Longxing Temple, and Longzang Palace, have been gazetted as heritage buildings by the government [34], as shown in Fig 3.

Longxing Ancient Town was chosen for this study because it is strategically located in the most popular tourist city in China. Like many other historical and cultural towns, Longxing Ancient Town has not attracted enough attention from tourists even though it has excellent tourism resources. This study took the recognition of the cultural value of heritage buildings as an entry point and tried to sort out the concerns of stakeholders in the process of heritage conservation.

### 3.3. Data collection and analysis of interviews

This study involved interviewing two categories of stakeholder: local shop owners and visitors. Before conducting the formal interviews, participants were verbally informed of the purpose of the interviews and assured them that their identity would remain strictly confidential. The researcher only commenced interviews upon obtaining participants’ consent; should a participant decline, the researcher proceeded to the next interviewee.

#### 3.3.1. Face-to-face interviews.

Local communities are frequently regarded as pivotal stakeholders in heritage tourism, heritage valuation, and cultural heritage management [35]. In 2002, as UNESCO commemorated the 30th anniversary of the World Heritage Convention, the Budapest Declaration on World Heritage underscored its significance, asserting, “It is imperative to facilitate the active involvement of local communities in the identification, conservation, and management of World Heritage properties at all levels”. Consequently, there is a mounting interest, particularly within the heritage sector, in engaging communities in the identification of heritage values within their localities [29].

The interviewees were selected from 152 shop owners in Longxing Ancient Town using a simple random sampling method. The interviews were concluded once saturation was reached. The researcher anticipated that interviewing 10–12 shop owners would achieve this. The criterion was that when the amount of new information obtained from two additional interviews was less than 5% of the total information collected from the first four interviews, the study was considered to have reached coding saturation [36]. The shops were divided into 10 groups, including 8 groups of 15 shops and 2 groups of 16 shops. Numbered paper balls were used to randomly select one interviewee from each group and another alternate interviewee to avoid filling in if the interviewee could not complete the interview or saturation was not reached after interviewing 10 interviewees. The interview outline was tailored for the local shop owners in advance. The interviews were conducted in June 2025 in Longxing Ancient Town, and each session lasted for about one hour. The entire interview process was conducted by two people; one person guided the conversation, and the other recorded the interview.

The purpose of the interview was to collect the shop owners’ views on the cultural value of heritage buildings. The interview questions included the town’s visitor profile, shop operations, the town’s attractions, the town’s architectural heritage, the cultural value of the heritage buildings, open-ended questions, and demographic information (S1 Appendix). Demographic questions were placed at the end of the interview because even when confidentiality is guaranteed, the disclosure of personal information would be considered an unwarranted invasion of privacy [36]. The tenth interview marked the point at which the phase of the interview reached saturation.

The respondents were predominantly male (60%), with an average age of 44 years. The majority (70%) had completed high school or less, and the annual shop income of the respondents exhibited a considerable range, with the majority falling between $15,000 and $45,000. As shown in Table 2.

**Table 2:** Demographic information of local shop owners.

Category	Description	Quantities
Gender	Male	6
	Female	4
Age	22 years old and below	0
	23–30 years old	0
	31–40 years old	3
	41–50 years old	4
	51–60 years old	3
	61 years old and above	0
Academic qualification	High school and below	7
	University or undergraduate	3
	Graduate or above	0
Annual shop income (I)	<$15,000	2
	$15,000 ≤ I < $25,000	4
	$25,000 ≤ I < $35,000	2
	$35,000 ≤ I < $45,000	1
	$45,000 ≤ I < $55,000	1
	≥$55,000	0

#### 3.3.2. Go-along interviews.

The go-along method is an in-depth qualitative interviewing method that involves the researcher accompanying individual respondents in their familiar surroundings. The role of the interviewer is frequently that of a tour guide, thereby enabling them to assume control of the activity and reducing the hierarchical relationship between interviewer and interviewee [37]. The conversations that ensue during this process can yield a wealth of data, given that the interviewees are situated in an environment with which they identify [38].

Another stakeholder is the visitors of Longxing Ancient Town. The CVES developed in this study ultimately serves the needs of visitors; consequently, their attitudes are indispensable and of paramount importance. Visitors who parked their cars and walked directly into the town were observed, especially those who carried travel items such as sun hats and travel bags. They were the focus of the “tracking”. Once the interviewers found that they needed to take pictures, they took the initiative to help them and then served as tour guides for free, following the interview outline designed in advance. In June 2025, the interviewers conducted go-along interviews with visitors in Longxing Ancient Town, and each interview lasted for 40–70 minutes. The interviewers developed a brief guide document to allow more spontaneous conversation; they could express issues that seemed irrelevant or could not be directly observed before [39], and they could also ask the interviewees about their views on the surrounding environment to better understand their perspectives. In addition, the interviewers took pictures that echoed the participants’ descriptions to facilitate discourse analysis and create a comprehensive picture story. For example, when the respondents pointed out a specific feature in the surrounding environment, they took pictures of said features.

The questions included familiarity with the town, plans for the day, knowledge of heritage buildings, the cultural value of the heritage buildings, open-ended questions, and demographic information (S2 Appendix). Once again, the interviewers left demographic questions for the end. The data were collated during the interview process, eventually reaching saturation at the end of the interview with 10 respondents.

The respondents were predominantly male (30%), with an average age of 39 years. The majority (80%) held a bachelor’s degree or above, and the majority of respondents reported an annual household income ranging from $15,000 to $55,000. The respondents’ occupations were diverse, including roles such as teachers, designers, salespersons, and managers. As shown in Table 3.

**Table 3:** Demographic information of visitors.

Category	Description	Quantities
Gender	Male	3
	Female	7
Age	22 years old and below	0
	23–30 years old	2
	31–40 years old	4
	41–50 years old	3
	51–60 years old	1
	61 years old and above	0
Academic qualification	High school and below	2
	University or undergraduate	5
	Graduate or above	3
Annual household income (I)	<$15,000	0
	$15,000 ≤ I < $25,000	2
	$25,000 ≤ I < $35,000	5
	$35,000 ≤ I < $45,000	1
	$45,000 ≤ I < $55,000	1
	≥$55,000	1
Occupation	Teachers	3
	Engineer	1
	Designer	2
	Programmer	1
	Salesperson	1
	Civil servants	1
	Business executives	1

#### 3.3.3. Analysis of interview data.

A comprehensive summary was prepared for all interview transcripts of the cultural value of heritage buildings, ensuring a clear presentation of all pertinent information and establishing a foundation for subsequent coding and analysis within the NVivo software. The responses provided by the interviewees were grouped according to pre-established themes, namely historic value, local/place value, aesthetic/artistic value, and scientific/technical value.

In the initial phase, the coding framework was defined. Several sub-nodes were included under each category to facilitate the analysis in greater detail. The sub-nodes under each theme were derived from specific interviews and common concepts in cultural heritage research. To illustrate, the historic value category comprises sub-nodes such as “architectural history”, “historical events”, and “historical contextual priorities”. The local/place value category encompasses “local characteristics”, “local cultural experience”, and “visitor interaction with the place”. The aesthetic/artistic value category includes “architectural aesthetics” and “combination of aesthetics and culture”. The scientific/technical values category incorporates “craftsmanship”, a “combination of technical maintenance and modernization”.

In the second phase, preliminary coding was carried out. Guided by the coding framework, each interview transcript was reviewed line by line and corresponded to a pre-determined coding node based on the interview content. First, overarching themes were identified and increasingly detailed coding was applied. Each piece of interview data can be coded on more than one occasion, according to the themes present in the content. In other words, a single piece of interview content may be associated with multiple nodes. To illustrate, if an interviewee makes a reference to the carving process and the utilization of materials in architectural design, this could be categorized under the node “technology: technical value of carving and materials in architecture” within the scientific/technical values. Concurrently, a mention of the preservation condition of the edifice could be assigned to the node “historic environment: preservation integrity of the historic value”. To ensure the objectivity and reproducibility of the qualitative coding, a systematic inter-coder agreement procedure was performed. After the lead researcher (H.H.) developed the initial coding framework based on the literature review, a second researcher (W.H.), who was not involved in the interview process, independently coded 30% of the randomly selected interview transcripts (6 out of 20 transcripts: three from local shop owners and three from visitors). The coding results from both researchers were compared using Cohen‘s Kappa coefficient, calculated via NVivo. The overall Kappa value was 0.84 (95% confidence interval: 0.78–0.90), indicating “almost perfect” agreement according to Landis and Koch’s (1977) benchmark [40]. Disagreements were primarily related to the boundary between ‘local value’ and ‘historic value’. All coding discrepancies were discussed and resolved in a consensus meeting, leading to the final refined coding framework. The remaining transcripts were then coded by the lead researcher using this finalised framework. This process ensured that the qualitative findings were not solely dependent on a single researcher’s subjective interpretation.

The next step was duplicate coding and corrections. Following the completion of the initial coding, a duplicate check was conducted to ensure the coding accuracy for each piece of data. Additionally, the initial coding was adjusted and optimized if necessary. Should new themes or sub-nodes be identified during the coding process—that is, values embodied in the data that were not captured in the initial framework—new nodes would be added as needed, or existing nodes modified as appropriate. This was done to ensure flexibility and accuracy in the coding system. For example, several interviewees discussed the “integration of modern technology in the restoration of buildings,” and this topic was not addressed in the preliminary coding. Thus, a new node, “integration of technical maintenance and modernization”, was created.

### 3.4. Data collection and analysis of FGD

#### 3.4.1. Implementation of FGD.

An FGD enables individuals to engage in collective reflection on a given theme, whilst simultaneously facilitating the emergence of a broader range of perspectives [41]. It is an established method of interpretation that can be used to elucidate the social and cultural values inherent in architectural objects [42]. The discussion was conducted with five experts in the field of heritage buildings: a manager in the ancient town, an operator in charge of the investment promotion of the town, an operator in charge of the master planning, design, and management of the town, an architect, and a government-recognized provincial expert of ancient building restoration techniques. Each expert was selected based on their high professional standard in either architecture or urban planning and their experience in the construction industry (10–20 years). Moreover, these experts cover virtually all key specialist fields relevant to heritage conservation.

In July 2025, an FGD was held at a tea garden in Longxing Ancient Town. Before the meeting, the facilitator delineated the objectives of the two phases of the discussion. The first phase entailed a review, adaptation, and supplementation of the refined CVES. The second phase involved the assignment of weights to the indicators in the scale. The second phase was carried out via email 72 hours after the first phase was completed, where the experts refined the corresponding content and returned the information. Before commencing the formal discussion, the moderator verbally assured participants that their identities would be kept confidential, a point with which all experts agreed. To establish a common basis for discussion, the moderator commenced the session with a two-minute slide presentation, which included photographs of heritage buildings in Longxing Ancient Town. This effectively conveyed the content and details of the physical characteristics of the heritage buildings. The descriptive statistics obtained from the interviews were used to initiate discussions with the panel of experts in seven areas. These included the structure and comprehensiveness of the scale, the applicability of the questions, the accuracy of the question formulation and the communication of the cultural values, the in-depth measurement of the experience of the cultural values, the reasonableness of the scoring methods, the applicability of the cultural values to the type of visitor, and other suggestions and improvements (S3 Appendix).

#### 3.4.2. Analysis of FGD data.

This study employed a fully manual approach, examining each discussion item individually. First, all of the participants’ views from the FGD were collated and categorized by content. Some elements were incorporated into the primary indicators established during the interview phase, while those that did not align with existing indicators were listed separately. The content of the primary and secondary indicators within the scale was then amended through additions and deletions. This process resulted in the development of a CVES for heritage buildings comprising five primary and 24 secondary indicators.

## 4. Generating a CVES for heritage buildings

The classification framework of cultural value was refined based on the literature from the past two decades and through face-to-face interviews, go-along interviews and FGD. Different data collection methods were employed for different stakeholders, and experts were invited to assign weights to the various indicators to further establish an evaluation scale, namely the CVES of heritage buildings with indicator weights.

### 4.1. CVES refined through interviews

The CVES, which was developed through the analysis of interview transcripts and subsequent refinement, is presented in Table 4. This scale comprises four major categories and 42 subcategories.

**Table 4:** CVES refined through interviews.

Dimensions	No.	Description	Score				
Local/place value	Ua1	The architecture of the old town blends well with the local natural environment.	1	2	3	4	5
	Ua2	I think the architecture of the ancient town blends well with the surrounding natural environment to form a unique landscape.	1	2	3	4	5
	Ua3	The architecture of the ancient town can reflect the characteristics of the Bayu culture well.	1	2	3	4	5
	Ua4	I experienced the rich Bayu culture during my visit to the ancient town.	1	2	3	4	5
	Ua5	Regular maintenance is essential to protect the cultural value of the ancient town.	1	2	3	4	5
	Ua6	I think daily building maintenance can effectively enhance the cultural value of the ancient town.	1	2	3	4	5
	Ua7	I am satisfied with the cultural atmosphere of the ancient town, which meets my expectations.	1	2	3	4	5
	Ua8	The old town can provide visitors with an immersive cultural experience.	1	2	3	4	5
	Ua9	The cultural architecture of the old town can attract different types of visitors.	1	2	3	4	5
	Ua10	I think the architecture of the old town is attractive to both culture lovers and ordinary tourists.	1	2	3	4	5
Scientific/technical value	Ub1	The building materials of the ancient town demonstrate a high level of craftsmanship and technology.	1	2	3	4	5
	Ub2	The materials and craftsmanship of the buildings are an important reflection of the cultural value of the ancient town.	1	2	3	4	5
	Ub3	The carvings and details in the buildings show a high level of craftsmanship.	1	2	3	4	5
	Ub4	The carving art and building materials of the ancient town make me feel the mastery of the craftsmen’s skills.	1	2	3	4	5
	Ub5	The restoration work of the buildings needs to rely on advanced technology to maintain their original appearance.	1	2	3	4	5
	Ub6	Modern technology plays an important role in the maintenance of buildings in the ancient town.	1	2	3	4	5
	Ub7	The architectural restoration of ancient towns combines traditional craftsmanship and modern technology very well.	1	2	3	4	5
	Ub8	I think modern technology can be used to maintain the structure of buildings while preserving traditional culture.	1	2	3	4	5
	Ub9	Restoration techniques are very important in preserving the cultural heritage of ancient towns.	1	2	3	4	5
	Ub10	Architectural restoration techniques ensure the long-term preservation of buildings in ancient towns.	1	2	3	4	5
Historic value	Uc1	I have enough knowledge about the historical background of the ancient town’s architecture.	1	2	3	4	5
	Uc2	Through the visit, I have a better understanding of the architectural history of the ancient town.	1	2	3	4	5
	Uc3	The architecture of an ancient town reflects the culture and lifestyle of a particular historical period.	1	2	3	4	5
	Uc4	The historical background of the architecture adds more cultural significance to the ancient town.	1	2	3	4	5
	Uc5	I feel that the architecture of the ancient town has a deep connection with the local history and culture.	1	2	3	4	5
	Uc6	As a national cultural heritage, the architecture of the ancient town has a great attraction to me.	1	2	3	4	5
	Uc7	I think the status of the ancient town as a cultural heritage adds to the value of its visit.	1	2	3	4	5
	Uc8	Historical background is the main reason for my interest in the architecture of ancient towns.	1	2	3	4	5
	Uc9	The historical background of an ancient town occupies a central position in the evaluation of its cultural value.	1	2	3	4	5
	Uc10	The architecture of old towns is closely related to important historical events.	1	2	3	4	5
Aesthetic/artistic value	Ud1	The architectural design style and color scheme of the old town are very harmonious and have an aesthetic value.	1	2	3	4	5
	Ud2	The design styles and color choices of the buildings enhance the artistic appeal of the ancient town.	1	2	3	4	5
	Ud3	The exterior design of the buildings in the ancient town has unique artistry and is in harmony with the surrounding environment.	1	2	3	4	5
	Ud4	The exterior design of the buildings complements the overall atmosphere of the ancient town.	1	2	3	4	5
	Ud5	The aesthetic combination of the buildings and the natural environment of the ancient town is impressive.	1	2	3	4	5
	Ud6	I believe that the integration of architecture and environment enhances the overall aesthetic appeal of the ancient town.	1	2	3	4	5
	Ud7	The aesthetic design of historical buildings is one of the highlights of the old town.	1	2	3	4	5
	Ud8	The architecture of the ancient town attracts a large number of tourists because of its historical aesthetics.	1	2	3	4	5
	Ud9	The design style of the buildings blends well with the cultural stories, adding depth to the visiting experience.	1	2	3	4	5
	Ud10	I think the architectural style of the ancient town reflects its cultural background very well.	1	2	3	4	5
	Ud11	The visual design of the architecture of the ancient town made me understand more about its cultural value.	1	2	3	4	5
	Ud12	The aesthetic design of the architecture allowed me to feel the cultural depth behind the ancient town.	1	2	3	4	5

Notes: 1 for very unconcerned, 2 for not concerned, 3 for neutral, 4 for concerned, and 5 for very concerned.

### 4.2. CVES Optimized through FGD

Based on the feedback from the FGD, the dimensions and specific topics were expanded and adjusted to enhance the scale’s comprehensiveness, applicability, and accuracy.

First, the “Sustainability Value” dimension was added to be more relevant to the theme of the study. Heritage buildings not only carry historical and artistic significance but also form a symbiotic relationship with the neighboring community and ecosystem. This dimension helps to evaluate the performance of heritage buildings in terms of cultural heritage and resource utilization, responds to the trend of harmonious development of heritage buildings preservation and culture, reflects the long-term value of heritage buildings in modern society, and addresses the public’s concern for sustainable cultural development.

In terms of the title design, the original title was made more specific and popularized, especially in terms of “scientific value” and “historic value”, and added descriptions of building materials and craftsmanship details to make the title reflect the technical and historical features of the heritage building more precisely. This kind of specific expression not only enhances the professionalism of the topic but also helps visitors to understand and evaluate the craftsmanship and historical connotations of heritage buildings more easily. At the same time, some terms were simplified and more understandable language was adopted to enhance the scale’s suitability to general tourists, and to expand the scale’s applicability to a wider range of people.

In addition, the description of the primary indicators was further simplified: aesthetic value and artistic value have similar connotations and were thus uniformly described as aesthetic value; similarly, local value and place value were uniformly described as local value, scientific value, and technical value were also uniformly described as scientific value.

Through these adjustments, the scale was designed to be more multidimensional and specific, and can be adapted to the understanding level of different visitor types, to comprehensively and accurately evaluate the cultural value of heritage buildings. The optimized scale is shown in Table 5.

**Table 5:** CVES optimized through FGD.

Dimensions	No.	Description	Score				
Local value	U11	The building is well integrated into the local natural environment.	1	2	3	4	5
	U12	The building co-exists harmoniously with its surroundings, creating a holistic landscape.	1	2	3	4	5
	U13	The building is a good reflection of the local culture.	1	2	3	4	5
	U14	Local materials were largely used in the building.	1	2	3	4	5
	U15	The routine maintenance of the building effectively ensures the local character of the building.	1	2	3	4	5
Scientific value	U21	The craftsmanship of building construction is an important expression of cultural values.	1	2	3	4	5
	U22	The carvings and details in the building demonstrate a high level of craftsmanship.	1	2	3	4	5
	U23	Modern technology plays an important role in building maintenance.	1	2	3	4	5
	U24	The building restoration is a good combination of traditional craftsmanship and modern technology.	1	2	3	4	5
Historic value	U31	The tour gave me a better understanding of the history of the building.	1	2	3	4	5
	U32	Architecture reflects the culture and lifestyle of a particular historical period.	1	2	3	4	5
	U33	The historical context of the buildings adds more cultural significance to the old town.	1	2	3	4	5
	U34	The building of the old town is deeply connected to the local history and culture.	1	2	3	4	5
	U35	The building is strongly associated with important historical events.	1	2	3	4	5
	U36	The building’s status as a cultural heritage site adds to its value as it is visited.	1	2	3	4	5
Aesthetic value	U41	The building design is very harmonious in style and color scheme and has an aesthetic value.	1	2	3	4	5
	U42	The design style and color choices of the buildings enhance the artistic appeal of the town.	1	2	3	4	5
	U43	The appearance of the building is uniquely artistic and in harmony with its surroundings.	1	2	3	4	5
	U44	The aesthetic design of the building gave me a sense of the cultural depth behind the town.	1	2	3	4	5
	U45	The exterior of the building is attractive to both cultural enthusiasts and casual visitors.	1	2	3	4	5
Sustainability value	U51	The building co-exists harmoniously with its surroundings without damaging the overall landscape.	1	2	3	4	5
	U52	The conservation of the building minimizes damage to the original structure of the building.	1	2	3	4	5
	U53	The conservation of buildings promotes the continuity of architectural culture.	1	2	3	4	5
	U54	The cultural preservation of buildings is key to the sustainability of local culture.	1	2	3	4	5

Notes: 1 for very unconcerned, 2 for not concerned, 3 for neutral, 4 for concerned, and 5 for very concerned.

### 4.3. The iteration of the CVES

**Fig 4:**
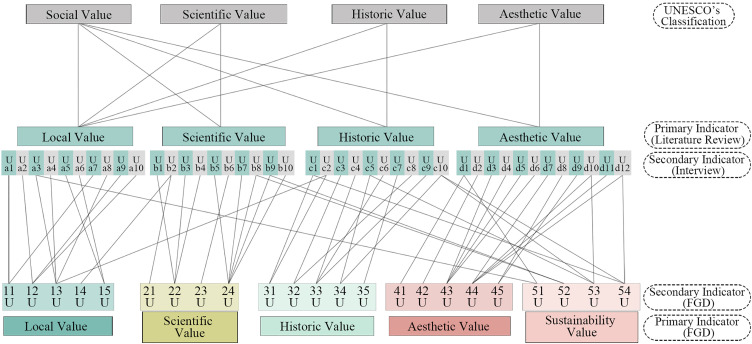
Iteration of the indicators based on cultural value classification.

This study focuses on the CVES. The origins of research in this field can be traced back to UNESCO’s 1972 classification of cultural values. Subsequently, researchers redefined the scale’s primary indicators based on a review of the literature on cultural value classification studies. Secondary indicators were then defined through interviews with a range of stakeholders from non-expert groups. FGD were then conducted with experts, who refined the primary and secondary indicators of the scale comprehensively. The Indicator Iteration Flowchart illustrates the iterative development process of the CVES (Fig 4).

### 4.4. Determination of indicator weights through AHP

#### 4.4.1. Basic steps of the AHP.

**Fig 5:**
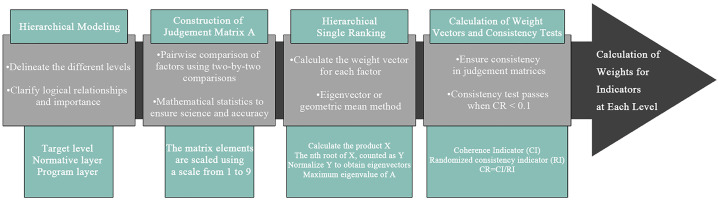
Flowchart of the steps of AHP implementation.

Determining the weights of each indicator using the Analytical Hierarchy Process (AHP) consists of four basic steps: 1) construction of a hierarchical model, 2) construction of judgment matrix A, 3) hierarchical single ranking, and 4) calculation of weight vectors and consistency test. Fig 5 presents the flowchart of AHP implementation, adopted from the book Analytic Hierarchy Process and Its Extensions by Ishizaka [43].

Based on multiple rounds of expert consultation using the Delphi method, the judgment matrix A was constructed. As the largest characteristic root of the judgment matrix is influenced by its constituent elements, the non-consistency of the judgment matrix A becomes more pronounced as the value of n increases. To quantify this degree of inconsistency, the Consistency Index (CI) is employed. A lower CI value indicates a greater degree of consistency within the judgment matrix, whereas a higher CI value indicates a lesser degree of consistency. In particular, the eigenvector corresponding to the largest eigenvalue is employed as the weight vector for each factor. As the degree of inconsistency increases, so does the judgment error. Accordingly, the extent of inconsistency in the judgment matrix A can be quantified by calculating the CI value to assess its accuracy and reliability. CI is calculated as follows:


$$\begin{array}{c} CI=\frac{\lambda_{max}-n}{n-1} \end{array}$$ (1)

When CI is equal to 0, it indicates that the judgement matrix has complete consistency; when CI is close to zero, it indicates that the consistency is more satisfactory. A larger CI value indicates a more serious inconsistency. To better assess the size of CI, the random consistency index (RI) is introduced, calculated as follows:


$$\begin{array}{c} RI=\frac{CI_{\text{1}}+CI_{\text{2}}+\text{\textbackslash{}ldots}+CI_{n}}{n} \end{array}$$ (2)

The Random Index (RI) is an important parameter used to assess the consistency of a judgment matrix, and it is closely related to the order of the judgment matrix. Usually, a larger order of the judgment matrix denotes a greater possibility of random deviation in consistency. As shown in Table 6, the corresponding RI values of judgment matrices of different orders are different.

**Table 6:** Mean random consistency indicator RI values.

n step	3	4	5	6	7	8	9	10	11	12
RI value	0.52	0.89	1.12	1.26	1.36	1.41	1.46	1.49	1.52	1.54

To get a more comprehensive and objective consistency evaluation, the CI was compared with the RI to derive the CR value. Normally, when CR < 0.1, the judgment matrix is considered to have passed the consistency test, i.e., it has satisfactory consistency; if CR ≥ 0.1, it indicates that the consistency of the judgment matrix is poor and needs to be adjusted and corrected to improve consistency.


$$\begin{array}{c} CR=\frac{CI}{RI} \end{array}$$ (3)

#### 4.4.2. Calculation of indicator weights.

After the FGD, the principal components of the scale were delineated, which were subsequently evaluated by the five experts participating in the discussion, the geometric mean was used to aggregate the five individual pairwise comparison matrices into a single group matrix (S4 Appendix). Based on the scoring results, a criterion-level judgment matrix and weighting results were established, as shown in Table 7.

**Table 7:** Table of weights for primary indicators.

	Local value (A1)	Scientific value (A2)	Historic value (A3)	Aesthetic value (A4)	Sustainability value (A5)	Wi	CR
**Local value (A1)**	1	1 4/9	1 1/3	1 3/7	1 2/5	0.2583	0.0055
**Scientific value (A2)**	5/7	1	2/3	1 3/8	4/5	0.1730	
**Historic value (A3)**	3/4	1 4/9	1	1 3/8	1 1/6	0.2201	
**Aesthetic value (A4)**	5/7	5/7	5/7	1	4/5	0.1541	
**Sustainability value (A5)**	5/7	1 1/4	6/7	1 1/4	1	0.1945	

The CR value of the primary indicators A1–A5 is 0.0055, which is less than 0.1 and thus meets the consistency test standard. Furthermore, the data are deemed to be reasonable. Subsequently, the judgment matrix and weighting calculation of the secondary indicators, obtained through the scoring of the secondary indicators by five experts, indicates that the CR value of local value (A1) is 0.0008, the CR value of scientific value (A2) is 0.0021, the CR value of historic value (A3) is 0.0036, the CR value of aesthetic value (A4) is 0.0041, and the CR value of sustainable value (A5) is 0.0030. The CR values of A1 to A5 are less than 0.1, thus meeting the consistency test criteria and demonstrating the reasonableness of the data. The weighted values of the composite indicators are presented in Table 8.

**Table 8:** Composite indicator weights.

Primary indicators	Primary indicator Weights	Secondary Indicators	Secondary Indicator Weights	Composite Indicator Weights
Local value (A1)	0.2583	U11	0.2280	0.0589
		U12	0.1642	0.0424
		U13	0.2481	0.0641
		U14	0.1647	0.0425
		U15	0.1950	0.0504
Scientific value (A2)	0.1730	U21	0.3059	0.0529
		U22	0.2642	0.0457
		U23	0.2381	0.0412
		U24	0.1919	0.0332
Historic value (A3)	0.2201	U31	0.1637	0.0360
		U32	0.1754	0.0386
		U33	0.2104	0.0463
		U34	0.1249	0.0275
		U35	0.1881	0.0414
		U36	0.1375	0.0303
Aesthetic value (A4)	0.1541	U41	0.1608	0.0248
		U42	0.2160	0.0333
		U43	0.2044	0.0315
		U44	0.1580	0.0243
		U45	0.2609	0.0402
Sustainability value (A5)	0.1945	U51	0.1983	0.0386
		U52	0.3068	0.0597
		U53	0.2728	0.0531
		U54	0.2221	0.0432

## 5. Validation and ethics statement

Before presenting the validation results, the reliability of the qualitative coding process (inter-coder Kappa = 0.84) and the transparency of the AHP expert aggregation (see S4 Appendix for raw individual matrices) together support the robustness of the CVES developed in this study. Subsequently, the researchers conducted a questionnaire survey based on the scale. During the testing phase, this study mainly addressed the following issues: (1) testing the operability of the questionnaire for distribution and retrieval; (2) testing the reasonableness of the question set in the questionnaire; and (3) testing the reliability and consistency of the relevant information. Only when all three aspects mentioned above are proven to be feasible will the scale be deemed suitable for quantifying the cultural value of heritage buildings.

### 5.1. Data collection during the testing phase

Before the survey, the researcher carefully designed the questionnaire to collect basic information about the respondents and their perceptions of the cultural value of the heritage buildings, which relied on the scale questions derived from the cultural value evaluation indicators. In January 2026, the researcher planned to randomly distribute the questionnaire to 200 visitors. Among the visitors in Longxing Ancient Town, visitors were randomly selected within defined field constraints, i.e., four streets with a relative concentration of visitors were chosen to arrange a researcher to randomly distribute the electronic questionnaires to the nearest visitor (accompanied by clear communication and small cultural gifts), and if the target visitor was unwilling or unable to participate in the survey, he/she was replaced by another visitor who was relatively close to the target visitor. Each time a questionnaire was distributed, the researchers informed the participants that their information would be treated confidentially. The basic personal details provided were used solely for statistical purposes, in order to characterize the sample, and do not involve any specific personal information. Once consent has been obtained, participants are required to scan a QR code to complete the questionnaire. After distributing 50 questionnaires per person across the four streets, distribution ceased, with a total of 200 questionnaires issued. A total of 192 valid questionnaires were collected from visitors in this way. The questionnaires were based on the physical characteristics of the five heritage buildings in Longxing Ancient Town (overall characteristics, characteristics at close range, and interior characteristics), and the researcher took three groups of photographs for each building to show the different physical characteristics of the building, with a total of 15 groups of photographs, and the respondents scored the indicators of a group of photographs of one heritage building at a time, and each respondent completed the scoring procedure 15 times. The average score for each secondary indicator was then calculated for each heritage building. Subsequently, the average score for each primary indicator was derived across the five heritage buildings of Longxing Ancient Town. The characteristics of these respondents are shown in Table 9.

**Table 9:** Statistics on sample characteristics.

Type of information	Options	Frequency	Percentages (%)	Cumulative percentage(%)
Gender	Male	108	56.25	56.25
	Female	84	43.75	100.00
Age	22 years and under	11	5.73	5.73
	23-30 years	46	23.96	29.69
	31-40 years	58	30.21	59.90
	41-50 years	50	26.04	85.94
	51-60 years	20	10.42	96.36
	61 years and over	7	3.64	100.00
Academic qualifications	High school and below	92	47.92	47.92
	Undergraduate	81	42.19	90.11
	Graduate students and above	19	9.89	100.00
Occupational category	Practitioners in heritage conservation, urban planning, architecture, and other related professions	35	18.23	18.23
	Practitioners of other professions	157	81.77	100.00
**Add up the total**		192		

During the testing phase, researchers evaluated five different dimensions of cultural value of heritage buildings (local value, scientific value, historic value, aesthetic value, and sustainability value). Respondents’ evaluation of each indicator of cultural value of heritage buildings was based on a Likert scale (5-point scale), with 1 for very unconcerned, 2 for not concerned, 3 for neutral, 4 for concerned, and 5 for very concerned. The average score of each secondary indicator was calculated, and then the score of each primary indicator was summarized and calculated, as shown in Table 10. The results showed that the mean value of each dimension ranges from 3.144 to 3.396, the median fluctuates from 3.200 to 3.800, and the standard deviation is in the range of 0.834 to 1.092, which indicates that the data distribution has a certain degree of dispersion. The highest and lowest scores of each dimension also showed differences, reflecting the diversity of evaluation objects in different value dimensions. These basic indicator data provided an important reference for subsequent in-depth analysis.

**Table 10:** Descriptive analysis.

Value categories	Sample size	Minimum value	Maximum value	Average value	Standard deviation	Median
Local value	192	1.200	4.400	3.144	0.834	3.200
Scientific value	192	1.500	4.750	3.250	0.985	3.500
Historic value	192	1.330	5.000	3.300	1.062	3.415
Aesthetic value	192	1.800	4.800	3.396	0.957	3.800
Sustainability value	192	1.250	4.750	3.195	1.092	3.500

### 5.2. Reliability analysis

Based on the results of Cronbach’s reliability analysis, the alpha coefficients corresponding to the dimensions of local value, scientific value, historic value, aesthetic value, and sustainability value are 0.901, 0.871, 0.941, 0.906, and 0.910, which are all significantly higher than the critical value of 0.8. As shown in Table 11. This result clearly showed that the internal consistency of the measurement items of each value dimension is at a high level, which meant that these measurement items can stably and effectively reflect the characteristics of heritage buildings in the corresponding value dimension. At the same time, most of the Corrected Item-Total Correlation (CITC) values under each value dimension reached 0.7 and above, which further strongly confirmed the close correlation between the measurement items and the validity of the measurement tool. In summary, the reliability analysis demonstrates that the proposed scale accurately measures the multi-dimensional cultural value of heritage buildings in ancient towns. The high reliability coefficients (all > 0.87) indicate strong internal consistency. This finding establishes a solid and reliable data foundation for subsequent analyses, enabling in-depth investigation and advancing research in this field.

**Table 11:** Cronbach’s reliability analysis.

Indicators	Name	Corrective Item-Total Correlation(CITC)	Item with deleted α coefficient	Cronbach’s α coefficient
Local value	U11	0.828	0.863	0.901
	U12	0.719	0.888	
	U13	0.779	0.875	
	U14	0.713	0.890	
	U15	0.755	0.880	
Scientific value	U21	0.854	0.787	0.871
	U22	0.634	0.868	
	U23	0.702	0.843	
	U24	0.776	0.826	
Historic value	U31	0.910	0.920	0.941
	U32	0.848	0.927	
	U33	0.777	0.935	
	U34	0.885	0.922	
	U35	0.781	0.935	
	U36	0.754	0.938	
Aesthetic value	U41	0.829	0.873	0.906
	U42	0.742	0.890	
	U43	0.783	0.881	
	U44	0.787	0.880	
	U45	0.702	0.897	
Sustainability value	U51	0.889	0.853	0.910
	U52	0.835	0.870	
	U53	0.709	0.913	
	U54	0.784	0.888	

### 5.3. Validity analysis

According to the results of Kaiser-Meyer-Olkin Measure (KMO) and Bartlett’s test analysis, the KMO value is 0.745, which indicates that the correlation between variables was moderate and suitable for factor analysis. Meanwhile, the approximate chi-square value of Bartlett’s test of sphericity was 972.238, with 276 degrees of freedom, and the p-value reached a significant level (p = 0.000), which rejected the hypothesis that the variables were independent of each other, further supporting the suitability of the data for factor analysis. As shown in Table 12.

**Table 12:** KMO and Bartlett’s tests.

KMOvalue		0.745
Bartlett’s test of sphericity	Approximate chi-square	972.238
	df	276.000
	p-value	0.000

### 5.4. Variance explained by principal components

According to the results of the analysis of the table of variance explained, it can be seen that the first five rotated principal components explained 77.678% of the variance cumulatively, of which the first principal component explained 19.879% of the variance, the second explained 15.711% of the variance, the third explained 15.336% of the variance, the fourth explained 12.699% of the variance, and the fifth principal component explained 14.053% of the variance. As shown in Table 13. This indicates that these five principal components represent most of the information of the original data well.

**Table 13:** Table of variance explained.

No.	Eigenvalue			Variance explained before rotation			Variance explained after rotation		
	Eigenvalue	Variance explained%	Cumulative%	Eigenvalue	Variance explained%	Cumulative%	Eigenvalue	Variance explained%	Cumulative%
1	8.629	35.955	35.955	8.629	35.955	35.955	4.771	19.879	19.879
2	3.602	15.008	50.963	3.602	15.008	50.963	3.771	15.711	35.590
3	2.491	10.378	61.340	2.491	10.378	61.340	3.681	15.336	50.926
4	2.380	9.917	71.257	2.380	9.917	71.257	3.048	12.699	63.625
5	1.541	6.421	77.678	1.541	6.421	77.678	3.373	14.053	77.678
6	0.685	2.855	80.533	0.685	2.855	80.533			
7	0.598	2.492	83.026	0.598	2.492	83.026			
8	0.524	2.185	85.211	0.524	2.185	85.211			
9	0.489	2.039	87.249	0.489	2.039	87.249			
10	0.462	1.925	89.175	0.462	1.925	89.175			
11	0.389	1.620	90.795	0.389	1.620	90.795			
12	0.373	1.554	92.349	0.373	1.554	92.349			
13	0.293	1.223	93.572	0.293	1.223	93.572			
14	0.257	1.071	94.643	0.257	1.071	94.643			
15	0.243	1.015	95.658	0.243	1.015	95.658			
16	0.228	0.951	96.609	0.228	0.951	96.609			
17	0.174	0.724	97.333	0.174	0.724	97.333			
18	0.155	0.646	97.979	0.155	0.646	97.979			
19	0.139	0.579	98.557	0.139	0.579	98.557			
20	0.107	0.446	99.004	0.107	0.446	99.004			
21	0.089	0.369	99.373	0.089	0.369	99.373			
22	0.075	0.311	99.684	0.075	0.311	99.684			
23	0.045	0.189	99.873	0.045	0.189	99.873			
24	0.030	0.127	100.000	0.030	0.127	100.000			

### 5.5. Results of rotated factor loading analysis

The results of the table analysis of the rotated factor loading coefficients showed that the five factors have different degrees of explanatory power for the 24 descriptive items related to cultural values. The factors reflected significant loading differences in terms of natural integration, cultural reflection, material use, maintenance value, and artistic attractiveness of the buildings, and had a high degree of commonality, indicating that these factors can capture and explain the multidimensional characteristics of heritage buildings in ancient towns more comprehensively. As shown in Table 14. This result contributed to an in-depth understanding of the cultural value of heritage buildings in ancient towns and their conservation importance.

**Table 14:** Table of rotated factor loadings.

Name	Factor loadings					Communalities (Common Variance)
	Indicator 1	Indicator 2	Indicator 3	Indicator 4	Indicator 5	
U11	−0.043	0.899	0.022	0.030	0.174	0.842
U12	0.107	0.811	0.044	0.162	0.016	0.698
U13	0.038	0.866	0.100	0.080	0.020	0.769
U14	0.218	0.756	0.224	−0.131	0.197	0.725
U15	0.053	0.782	0.011	0.049	0.374	0.756
U21	0.195	0.037	0.082	0.898	0.141	0.872
U22	0.193	−0.125	0.190	0.724	0.171	0.643
U23	0.283	0.217	0.025	0.740	0.176	0.706
U24	0.212	0.124	0.151	0.806	0.197	0.771
U31	0.925	0.075	0.104	0.178	−0.001	0.903
U32	0.882	0.072	0.139	0.094	0.080	0.817
U33	0.789	0.171	0.275	0.150	−0.025	0.751
U34	0.879	0.099	0.119	0.171	0.181	0.859
U35	0.795	0.089	0.149	0.200	0.082	0.708
U36	0.771	−0.116	0.168	0.216	0.233	0.737
U41	0.067	0.136	0.860	0.088	0.215	0.817
U42	0.257	0.124	0.771	0.201	0.075	0.721
U43	0.277	−0.071	0.854	0.112	0.026	0.824
U44	0.147	0.088	0.852	0.091	0.055	0.767
U45	0.129	0.143	0.704	0.004	0.444	0.729
U51	0.111	0.240	0.162	0.095	0.883	0.884
U52	0.215	0.284	0.140	0.098	0.829	0.844
U53	0.062	0.108	0.206	0.361	0.711	0.693
U54	0.057	0.060	0.089	0.265	0.848	0.804

After testing, the operability of the questionnaire met the standard, the reasonableness of the questions set in the questionnaire met the requirements, and the reliability and consistency of the related information passed the test. In addition, during the questionnaire survey, the respondents did not give feedback on the problems related to the content of the questionnaire, which indicated that the content of this questionnaire is clearly expressed and could be fully applied to the study, and it can be applied to the questionnaire survey stage.

### 5.6. Ethics statement

This study involved face-to-face semi-structured interviews with 10 local shop owners, go-along interviews with 10 visitors, a FGD with five experts, and an online questionnaire completed by 192 visitors to the historical and cultural town. All participants were adults (aged 18 years or older). No personally identifiable information was collected.

Prior to participation, all interviewees and expert discussion participants were verbally informed about the purpose of the study, its voluntary nature, their right to decline or withdraw at any time, and the measures taken to ensure confidentiality and anonymity. Verbal informed consent was obtained before data collection began. Given the minimal-risk nature of the study and its conduct in naturalistic field settings, written consent forms and audio recordings were not used in order to preserve participant anonymity and avoid disrupting natural interactions. The consent process was documented through contemporaneous researcher field notes confirming that informed verbal consent had been obtained. For the online questionnaire survey, participants were presented with an introductory statement outlining the study objectives, voluntary participation, and confidentiality measures. Informed consent was indicated by participants’ voluntary completion and submission of the questionnaire.

The authors’ affiliated institution does not maintain a formal Institutional Review Board or ethics committee for social science field studies. All procedures were conducted in accordance with internationally accepted ethical standards for research involving human participants and adhered to the principles of the Declaration of Helsinki.

## 6. Discussion

Since UNESCO published the categories of cultural value in 1972, scholars have consistently endeavored to expand and refine their concepts. However, the intangible and continuously changing nature of cultural value ultimately leads to diverse and overlapping discussions that can be confusing and misleading [44]. The interrelated nature of the concepts of culture and value, coupled with the inherent contradictions between them, presents a significant challenge for the categorization of cultural value [45]. Previous research has integrated conceptually similar categories; for example, Stephenson’s (2005) discussion of the relationships and practices that describe history, science, aesthetics, and local values [29]. Silva and Roders (2012) used the term age, which is equivalent to historic value [12]. Yang (2012) discussed creative/innovative value, which is similar to aesthetic value [45]. Dhadphale (2017) used the terms storytelling and memory, which are similar to historic value [46]. Krajnik et al. (2022) discussed documentary as a comparable example of historic value [13]. In terms of evaluation frameworks relating to cultural values, BREEAM addresses this to some extent, although its focus remains limited. Although UNESCO/ICOMOS guidance has applied HIA within the heritage sector, it does not specifically evaluate cultural heritage values. Therefore, in terms of the framework’s comprehensiveness, this study has redefined and broadened the scope of local value beyond the existing framework, introducing a new category: sustainability value.

### 6.1. The “Wide” and “Narrow” concepts of local value

Local value can be defined as a value that is confined to a specific geographical area, thereby implying a certain degree of restriction (conceptually “narrow”), or as a category of heritage values that exists in cultural heritage across the globe, suggesting a broader scope (conceptually “wide”). Many scholars who discuss categories of cultural value describe the dual connotations of such values without generalizing them to local value. For example, the value of authenticity, as discussed by Throsby (2003), shared similarities with local value [11]. Similarly, the value of therapeutic rehabilitation, as outlined by Yang, could also be attributed to local value [45]. Dhadphale (2017) highlighted the importance of self-identity and group belonging, which could be seen as a manifestation of local value [46]. The distinctive characteristics of folklore and culture were described by Li, while the life culture of humans and nature reflected the connotation of local value [47]. In the present era, there is a growing emphasis on the excavation and evaluation of local culture, historical lineage, and architectural style in the context of heritage conservation [19]. Hwang (2014) termed this as “local culture-oriented urban regeneration”, which could be seen as a reflection of local value preservation [48]. In the interviews conducted for this study, one of the interviewees said, “The buildings are particularly local in character, and the layout, which is built on the mountain, is highly distinctive.” The construction of these edifices and the sentiments evoked are all manifestations of local value. Heritage buildings can serve as an emblem of the local cultural identity of the inhabitants [49,50]. It is therefore evident that the value of heritage buildings, which have played a pivotal role in human history and culture, should be recognized at the local level. This is based on the premise that local value is the primary consideration, given its intrinsic local qualities, and that it can convey the broader significance of such heritage. In evaluating the cultural value of heritage buildings, local value occupies a central position and is indispensable. Some of the achievements of past scholars in classifying cultural values have been incorporated into the concept of local values. Furthermore, following a critical analysis of these classifications by some scholars, researchers have also integrated them into the framework, thereby enriching its connotation.

### 6.2. The “New” and “Old” concepts of sustainability value

Sustainability was introduced by the United Nations in the 1980s and is still widely regarded as a fundamental concept in cultural heritage (conceptually “old”). However, it is challenging to accurately reflect the significance of cultural sustainability in the existing categorization framework of cultural value, as summarized in the systematic review. This concept is included in this study as a relatively new indicator (conceptually “new”). In the interviews, several visitors and local shop owners expressed their hope that the heritage buildings can be preserved for a long time and serve modern life better. In the FGD, the experts repeatedly mentioned the significance of emphasizing the sustainability of the built environment, especially from the perspective of the integration of the building and the environment. The importance of sustainability in heritage buildings, particularly in terms of integrating architecture and the natural environment, was underscored. Currently, scholars have discussed the application of sustainability value research in cultural heritage [51–55]. In addition, there is the potential for the development of a comprehensive sustainability-based cultural heritage evaluation methodology in alignment with the European Cultural Heritage Committee Program [56]. In the process of preserving heritage buildings, it is essential to consider whether the building in question co-exists harmoniously with its surrounding environment, whether it reduces damage to the original structure of the building, whether it promotes the continuity of architectural culture, and whether it ensures sustainable development of the local culture. These factors represent the core content of the sustainability value of heritage buildings. Furthermore, the “new” connotation of the “old” concept is likely to persist over the long term; this is the fundamental aspect of the sustainability value of heritage buildings. Building upon the concept of sustainable development proposed and continually refined by official organisations, this study has identified a new category: the sustainability value of heritage. This concept is characterized by its enduring dynamic nature and is indispensable within the framework for evaluating the cultural value of heritage buildings.

### 6.3. Differences in the weighting of indicators in the scale

**Fig 6:**
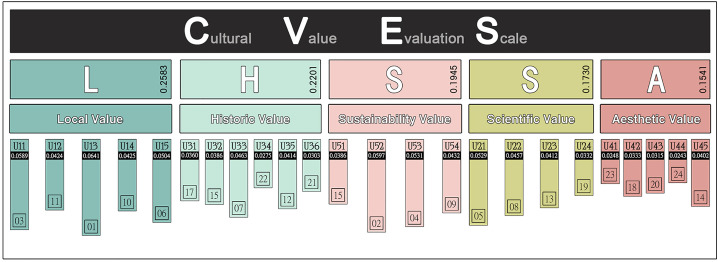
Cultural Value Evaluation Scale (CVES).

The evaluation of the cultural value of heritage buildings, generated through multiple perspectives of perception, is a complex process, with a multitude of connotations and significant variations in the assigned weights. In the field of urban planning, perception is defined as the process of gaining awareness and understanding of sensory information [57]. The process of value perception is, in fact, the process of perception itself. Consequently, the diversity of value is rooted in the diversity of values attributed to the subject [58]. In this study, the CVES of heritage buildings is presented as the LHSSA framework, comprising the following values: Local value, historic value, sustainability value, scientific value, and aesthetic value. The weights of the indicators are presented in descending order of the weights of the primary indicators (Fig 6). Of the 24 secondary indicators, the highest weighted, U13 (0.0641), has a weight 2.64 times greater than that of the lowest weighted, U44 (0.0243). The former pertains to local value, while the latter is concerned with aesthetic value. This distribution of weights is consistent with that observed for the primary indicators, which are ordered as follows: local value (0.2583), historic value (0.2201), sustainability value (0.1945), scientific value (0.1730), and aesthetic value (0.1541).

In terms of the content of the indicators, the experts unanimously expressed a preference for the display of local culture, while the aesthetic appeal of the building was not a significant area of interest. The content of local value is extensive and may be closely related to the concept of in situ conservation [59]. The second most significant primary indicator is historic value, due to the considerable number of concerns regarding historic value (six indicators), which is also the highest among all primary indicators. It is beyond question that urban cultural heritage buildings have an important historic value for both local and world civilizations [60]. The fact that sustainability value was weighted more heavily than scientific and aesthetic value indicates that the experts are more concerned with cultural continuity than with visually perceptible architectural entities. In other words, they believe that modern technology is sufficient to ensure the transmission of the scientific value and aesthetic value of architectural heritage. During the FGD, one expert emphasized the “value of long-term preservation of architectural heritage”, which is the sustainability value. The scientific value was accorded greater weight than the aesthetic value, given that several traditional craft techniques are in the hands of a limited number of individuals, even with designated “inheritors” [61], which makes it challenging to ensure the transfer of some traditional techniques.

In conclusion, the local value of architectural heritage in the future can be linked to the concept of in situ conservation, which has received the most attention amongst the expert community. Furthermore, the historic value of documenting people’s life course has always been the core value of architectural heritage, which can never be ignored. Then, the concept of sustainability value underscores the long-term impact of cultural influences. The inheritance of traditional craftsmanship is a key aspect of scientific value, while the aesthetic value of architecture is considered by experts to be relatively less important.

### 6.4. Similarities and differences among various stakeholders

In recent years, one of the most significant developments in the domain of evaluation has been the transition from a reliance on experts as authoritative sources to the incorporation of a diverse array of stakeholders, particularly community residents and visitors [62]. The feedback provided by the various stakeholders who participated in this study substantiated this assertion.

Local shop owners expressed the greatest interest in the historical context of the heritage buildings, which is why they chose to live or work in the area. When asked to rank possible values, the majority of shop owners ranked Historic Value first. One shop owner, for example, stated that “what attracts me the most is the rich history of the buildings”, and that “a lot of tourists will come for them”. For visitors, the majority also cited the historical significance of the heritage buildings as a primary motivation for their visit. One visitor stated, “These buildings have been around for hundreds of years, so I have to come and see them, and I find it interesting to learn how people lived in the past.” The second largest number of visitors came for the appearance of the buildings, which were described by one visitor as “evoking a sense of awe and appreciation for their cultural heritage”. This sentiment was echoed by another visitor who noted the intricate decorations and rich cultural connotations associated with them, stating, “I’ve seen those decorations in books before; each pattern has a rich cultural connotation, it’s so beautiful.” Additionally, experts have their preferences, with architects exhibiting a particular aversion to widely accepted postmodernism. This may be related to the fact that they accord the highest ranking to the local value and historic value of heritage buildings. The demolition of tangible heritage and reconstruction of replicas by some managers has resulted in the creation of numerous replica streets in historic districts [45], thereby severing the connection between architecture and history. Such actions disrupt the harmonious relationship between buildings and their surroundings [63], which is perhaps unsurprising given that managers tend to prioritize the aesthetic value of heritage buildings.

Consequently, the value ascribed to heritage buildings by the general public informs the strategies deployed for their conservation. This serves to reinforce the imperative for research into the evaluation of the cultural value of heritage buildings, and the scale summarized in this study is a welcome contribution to this field of inquiry.

### 6.5. Limitations and future research directions

The declining prominence of cultural studies in environmental science has resulted in the formulation of environmental policies that fail to adequately consider cultural value [64]. Cultural and non-use values are included with Ecosystem Services in all prominent typologies [10,26]. Nevertheless, in practice, there has been a paucity of attention devoted to cultural and non-use values within the growing body of empirical research on ecosystem services [65]. This field of study remains significantly underdeveloped due to the absence of a suitable scale for evaluating the cultural values of heritage buildings. The evaluation of value is regarded as a fundamental and pivotal stage in the processes of conservation and management [66]. To facilitate comparison and ranking of the value of heritage buildings, employing quantitative measurements of their value is necessary [56]. Given the inherent subjectivity and qualitative nature of classification criteria, the level of cultural significance of a given cultural heritage is often also determined based on a qualitative evaluation (UNESCO 2015a). This study developed CVES based on a single case study, which has certain limitations with regard to case selection. However, to mitigate this limitation, the researcher selected multiple stakeholders. Moreover, the data provided by the interviewees is more significant than the case selection itself. Beyond the extensive qualitative explorations currently underway [20,21,67,68], architects, academics, practitioners of traditional crafts, heritage conservation managers, and enthusiasts in the heritage field can quantitatively evaluate the cultural value of heritage buildings based on the CVES, which allows for scoring, comparing, and ranking the cultural value of the heritage building of individual buildings, communities, and even neighborhoods, and thus identifying which heritage buildings should be conserved and how to target them. Furthermore, the application of the CVES can be extended to all kinds of tangible and intangible cultural carriers of cultural heritage, which is of great significance and practical value for the recognition of heritage value and enrichment of the heritage value evaluation system.

## 7. Conclusion

Based on the classification framework for the cultural value of heritage developed over the past two decades, the categories of cultural value on heritage buildings were refined through interviews. Subsequently, experts were invited to conduct an FGD to optimize the scale. Finally, the indicators were assigned weights through the AHP, thus developing a CVES of heritage buildings for the first time, namely the LHSSA framework. Through suggestions from various stakeholders, the connotation of “local value” was expanded and the dimension of “sustainability value” was added. This resulted in the development of a scale comprising five primary indicators and 24 secondary indicators. The primary indicators, in descending order of weight, are local value, historic value, sustainability value, scientific value, and aesthetic value.

This study overcame the limitations of single-case research by engaging with multiple stakeholder groups and gathering valuable insights from each group in order to establish the CVES for the first time. This framework goes beyond evaluating individual buildings to enable the quantitative evaluation of cultural heritage value at community and neighborhood scales, significantly enriching the theoretical framework for heritage valuation. It provides practical guidance on specific valuation methodologies and informs the development of scientific heritage conservation strategies. Further optimization of the scale to accommodate intangible cultural carriers will enable its application to both tangible and intangible heritage objects. This offers comprehensive and far-reaching theoretical significance and practical value for recognising heritage value.

## Supporting information

S1 Appendix Appendix A. (DOCX)

S2 Appendix Appendix B. (DOCX)

S3 Appendix Appendix C. (DOCX)

S4 Appendix Appendix D. (DOCX)
