# Supplementary material for: An evaluation scale for the cultural value of heritage buildings
Source: PLoS One. 2026 Jun 23;21(6):e0350924. doi: 10.1371/journal.pone.0350924 (PMC13289924; doi:10.1371/journal.pone.0350924)
Supplement: S1 Appendix — (DOCX) [file pone.0350924.s001.docx]

Appendix A

#### Outline of a Face-to-Face Interview (local shop owners): Refinement of Scale Content

#### 1. Opening Topic and Pleasantries

- **Hey, business is good, what is your name?**
- **Do we have many visitors from outside the city? (Extension question: What types of visitors are there?)**

#### 2. Introduction to the Interviews

- **We are PhD students doing a study on improving the tourism appeal of ancient towns (showing student ID and research plan), and we would like to ask you some questions. Our small talk does not affect your reception of customers. I am also a customer (joking tone and shopping for some items at the same time).**

#### 3. Topics related to shop owners

- **When did you start opening a store here?**
- **Why did you choose to open a store here? (Extension question: Are you the owner or tenant?)**

#### 4. Topics related to the built environment

- **This town was rated as a historical and cultural town by the state in 2005. Has the management office promoted us as a national historical and cultural town? (Extension question: How does the historical and cultural towns influence shop operations?)**
- If we come here for the first time, what culture can we learn about here?
- **Which spots are the most worth visiting?**
- Why are they worth seeing?
- I am most interested in houses. I found out online that there are several heritage buildings here. Do you know them?

#### 5. Topics related to the cultural value

- **Because we are studying the content of architectural culture, some experts mentioned cultural value. Do you think the value of culture is great?**
- **In what aspects do you think the cultural value of a building is reflected? (Alternative question: What aspects do you pay attention to when visiting a building?)**
- What do you pay attention to in terms of the history of the building?
- What do you pay attention to in terms of the image of the building? (Replace aesthetic value)
- What do you pay attention to in terms of the craftsmanship of the building? (Replace scientific value)
- Every place has some buildings with local characteristics. What are the architectural characteristics here? (Replace local value)
- Other aspects of the interviewee’s attention (recorded in the interview)
- How do you rank the aspects you just mentioned according to your attention?
- The purpose of our research is to protect the cultural value of the building and enhance its cultural influence. Do you think this research is meaningful?
- Please give us some suggestions on the research on the cultural value of architecture. What else should be included?

#### 6. Supplementary Notes and Collection of Demographic Information

- **What you said was very helpful to us. We made a lot of notes. We want to include “***” in the report. Can we quote your name in the report?**
- **Finally, we need to record the interview situation. No personal information is involved. Please check the options in the form (quick demographic information multiple-choice questions).**
- Your gender (□Male □Female)
- Your age (□22 years old and below □23–30 years old □31–40 years old □41–50 years old □51–60 years old □61 years old and above)
- Your education (□High school and below □University or undergraduate □Graduate or above)
- Your Annual family income (□<$15,000 □$15,000 ≤ I < $25,000 □$25,000 ≤ I < $35,000 □$35,000 ≤ I < $45,000 □$45,000 ≤ I < $55,000 □≥$55,000)
