# Supplementary material for: An evaluation scale for the cultural value of heritage buildings
Source: PLoS One. 2026 Jun 23;21(6):e0350924. doi: 10.1371/journal.pone.0350924 (PMC13289924; doi:10.1371/journal.pone.0350924)
Supplement: S2 Appendix — (DOCX) [file pone.0350924.s002.docx]

Appendix B

#### Outline of a Go-Along Interview (visitors): Refinement of Scale Content

#### 1. Opening Topic and Pleasantries

- **Hey, do you need me to take a photo for you? (Observe and address when appropriate)**
- **It’s a pleasure to serve you. We are volunteers here.**

#### 2. Introduction to the Interviews

- **We are PhD students and are currently doing a research to improve the tourism competitiveness here (show student ID and research plan). We would like to hear your suggestions. Please follow your route. We can also provide explanations if you need (joking tone).**

#### 3. Topics related to visitors

- **Is this your first time here?**
- Why did you choose to come here? (Backup question: What aspects attracted you to come?)
- Where do you plan to visit today? (Backup question: Are there any target attractions?)
- What attractions attract you most here (what you knew before or feel now)?
- **We have several heritage buildings here. Did you know about them before?**

#### 4. Topics related to the cultural value

- **Our research is on architectural culture. Some experts mentioned cultural value. Do you think cultural value is great?**
- **In what aspects do you think the cultural value of a building is reflected? (Alternative question: What aspects do you pay attention to when visiting a building?)**
- What do you pay attention to in terms of the history of the building?
- What do you pay attention to in terms of the image of the building? (Replace aesthetic value)
- What do you pay attention to in terms of the craftsmanship of the building? (Replace scientific value)
- Every place has some buildings with local characteristics. What are the architectural characteristics here? (Replace local value)
- Other aspects of the interviewee’s attention (recorded in the interview)
- How do you rank the aspects you just mentioned according to your attention?
- The purpose of our research is to protect the cultural value of the building and enhance its cultural influence. Do you think this research is meaningful?
- Please give us some suggestions on the research on the cultural value of architecture. What else should be included?

#### 5. Supplementary Notes and Collection of Demographic Information

- **What you said was very helpful to us. We made a lot of notes. We want to include “***” in the report. Can we quote your name in the report?**
- **Finally, we need to record the interview situation. No personal information is involved. Please check the options in the form (quick demographic information multiple-choice questions).**
- Your gender (□Male □Female)
- Your age (□22 years old and below □23–30 years old □31–40 years old □41–50 years old □51–60 years old □61 years old and above)
- Your education (□High school and below □University or undergraduate □Graduate or above)
- Your occupation (Please fill in manually: )
- Your Annual household income (□<$15,000 □$15,000 ≤ I < $25,000 □$25,000 ≤ I < $35,000 □$35,000 ≤ I < $45,000 □$45,000 ≤ I < $55,000 □≥$55,000)
