# Supplementary material for: An evaluation scale for the cultural value of heritage buildings
Source: PLoS One. 2026 Jun 23;21(6):e0350924. doi: 10.1371/journal.pone.0350924 (PMC13289924; doi:10.1371/journal.pone.0350924)
Supplement: S3 Appendix — (DOCX) [file pone.0350924.s003.docx]

Appendix C

### Focus Group Discussion Outline: Scale Rationalization Discussion and Supplementation

#### 1. Structure and comprehensiveness of the evaluation scale

- **Discussion topic: Do you think that the four classifications of cultural values currently covered by the scale (local/place value, scientific/technical value, historic values, aesthetic/artistic value) are comprehensive, and are there other categories that should be considered?**
- **Supplementary question: Are there other categories or specific items that could enrich the existing scale to make the evaluation of cultural values more complete?**

#### 2. Applicability of the scales

- **Discussion topic:** Under each category of cultural values, do you think that the questions accurately reflect the characteristics of the category? Are there any unclear, over-generalized, or inappropriate statements?
- **Supplementary question:** What questions need to be revised or further explained to better reflect the real experiences of visitors and local shop owners?

#### 3. Scale formulation and indicators set for the cultural values

- **Discussion topic:** Is the presentation of each question clear and easy to understand, and can it accurately convey different cultural values (e.g. characteristics of the Bayu culture (local culture), craftsmanship skills, historical background)?
- **Supplementary question:** Is there a more appropriate formulation that would enhance the communication of cultural values and make the scale more practically instructive?

#### 4. Depth measurement of cultural value experiences

- **Discussion topic:** Do you think the scales are effective in measuring the depth and fulfillment of visitors’ cultural experiences? What items would better guide respondents in describing the quality of their experience?
- **Supplementary question:** In terms of measuring the depth of experience of cultural values, what specific questions can enhance the expression of this experience?

#### 5. Rationality of the way the scale is scored

- **Discussion topic:** Is the Likert scale (1–5) appropriate for cultural value evaluation? Are there alternative scales that would more clearly reflect respondents’ attitudes?
- **Supplementary question:** In terms of score refinement and understanding, is there a need to add notes or explanations to help respondents better select scores?

#### 6. Applicability of cultural values and visitor types

- **Discussion topic:** Are the scale questions adaptable to the needs and understandings of different types of visitors (e.g., cultural enthusiasts and ordinary tourists)? Is there a need to adapt some of the questions to be more relevant to the diverse visitor experience?
- **Supplementary question:** Is there a need to add supplementary questions to the scale for different types of visitors to increase the applicability of the evaluation?

#### 7. Other recommendations and improvements

- **Open discussion: Overall, what do you think could be added to or improved about the scale when evaluating cultural values? Do you have any other suggestions for specific questions, evaluation perspectives, or structures?**
