# Supplementary material for: An evaluation scale for the cultural value of heritage buildings
Source: PLoS One. 2026 Jun 23;21(6):e0350924. doi: 10.1371/journal.pone.0350924 (PMC13289924; doi:10.1371/journal.pone.0350924)
Supplement: S4 Appendix — (DOCX) [file pone.0350924.s004.docx]

Appendix D

### Individual Pairwise Comparison Matrices from Five Experts for AHP

****Aggregation Process:**** To derive the final group weights, the geometric mean was applied to the five individual matrices for each indicator set. The aggregated group matrix for the primary indicators is presented below in **Table D1**. This aggregated matrix was then used to calculate the final weights shown in Table 8 of the main manuscript. The same procedure was applied to all secondary indicator groups.

****Table D1. Aggregated group judgment matrix (Primary indicators) – derived by geometric mean****

|  | ****A1**** | ****A2**** | ****A3**** | ****A4**** | ****A5**** |
| --- | --- | --- | --- | --- | --- |
| A1 | 1.0000 | 1.4427 | 1.3510 | 1.4310 | 1.3977 |
| A2 | 0.6931 | 1.0000 | 0.6843 | 1.3797 | 0.8027 |
| A3 | 0.7402 | 1.4614 | 1.0000 | 1.3797 | 1.1761 |
| A4 | 0.6988 | 0.7248 | 0.7248 | 1.0000 | 0.8027 |
| A5 | 0.7155 | 1.2457 | 0.8503 | 1.2457 | 1.0000 |

****Eigenvalues and consistency:**** λmax = 5.0248, CI = 0.0062, CR = 0.0055.

****Note:**** The CR value of 0.0055 (< 0.1) indicates that the aggregated group judgment matrix has satisfactory consistency. The resulting weight vector for the primary indicators is: A1 (Local value) = 0.2583, A2 (Scientific value) = 0.1730, A3 (Historic value) = 0.2201, A4 (Aesthetic value) = 0.1541, A5 (Sustainability value) = 0.1945.

****1. Primary Indicators (5×5 matrices)****

The five primary indicators compared are:

A1: Local value

A2: Scientific value

A3: Historic value

A4: Aesthetic value

A5: Sustainability value

****Table D2. Pairwise comparison matrix – Expert 1 (Primary indicators)****

|  | ****A1**** | ****A2**** | ****A3**** | ****A4**** | ****A5**** |
| --- | --- | --- | --- | --- | --- |
| A1 | 1 | 4 | 2 | 5 | 3 |
| A2 | 1/4 | 1 | 1/5 | 3 | 1/3 |
| A3 | 1/2 | 5 | 1 | 5 | 2 |
| A4 | 1/5 | 1/3 | 1/5 | 1 | 1/4 |
| A5 | 1/3 | 3 | 1/2 | 4 | 1 |
| λmax = 5.2321 | CI = 0.0580 | CR = 0.0518 |  |  |  |

****Table D3. Pairwise comparison matrix – Expert 2 (Primary indicators)****

|  | ****A1**** | ****A2**** | ****A3**** | ****A4**** | ****A5**** |
| --- | --- | --- | --- | --- | --- |
| A1 | 1 | 5 | 3 | 6 | 4 |
| A2 | 1/5 | 1 | 1/4 | 4 | 1/2 |
| A3 | 1/3 | 4 | 1 | 6 | 3 |
| A4 | 1/6 | 1/4 | 1/6 | 1 | 1/4 |
| A5 | 1/4 | 2 | 1/3 | 4 | 1 |
| λmax = 5.3013 | CI = 0.0753 | CR = 0.0673 |  |  |  |

****Table D4. Pairwise comparison matrix – Expert 3 (Primary indicators)****

|  | ****A1**** | ****A2**** | ****A3**** | ****A4**** | ****A5**** |
| --- | --- | --- | --- | --- | --- |
| A1 | 1 | 1/4 | 1/2 | 1/6 | 1/3 |
| A2 | 4 | 1 | 4 | 1/4 | 2 |
| A3 | 2 | 1/4 | 1 | 1/6 | 1/2 |
| A4 | 6 | 4 | 6 | 1 | 4 |
| A5 | 3 | 1/2 | 2 | 1/4 | 1 |
| λmax = 5.1697 | CI = 0.0424 | CR = 0.0379 |  |  |  |

****Table D5. Pairwise comparison matrix – Expert 4 (Primary indicators)****

|  | ****A1**** | ****A2**** | ****A3**** | ****A4**** | ****A5**** |
| --- | --- | --- | --- | --- | --- |
| A1 | 1 | 1/4 | 1/2 | 1/5 | 1/3 |
| A2 | 4 | 1 | 3 | 1/3 | 2 |
| A3 | 2 | 1/3 | 1 | 1/6 | 1/4 |
| A4 | 5 | 3 | 6 | 1 | 4 |
| A5 | 3 | 1/2 | 4 | 1/4 | 1 |
| λmax = 5.2383 | CI = 0.0596 | CR = 0.0532 |  |  |  |

****Table D6. Pairwise comparison matrix – Expert 5 (Primary indicators)****

|  | ****A1**** | ****A2**** | ****A3**** | ****A4**** | ****A5**** |
| --- | --- | --- | --- | --- | --- |
| A1 | 1 | 5 | 3 | 6 | 4 |
| A2 | 1/5 | 1 | 1/4 | 5 | 1/2 |
| A3 | 1/3 | 4 | 1 | 6 | 3 |
| A4 | 1/6 | 1/5 | 1/6 | 1 | 1/3 |
| A5 | 1/4 | 2 | 1/3 | 3 | 1 |
| λmax = 5.3552 | CI = 0.0888 | CR = 0.0793 |  |  |  |

****2. Secondary Indicators****

****2.1. Local value (A1) – five secondary indicators (U11 to U15)****

****Table D7. Pairwise comparison matrix — Expert 1 (Local value A1)****

|  | ****U11**** | ****U12**** | ****U13**** | ****U14**** | ****U15**** |
| --- | --- | --- | --- | --- | --- |
| U11 | 1 | 5 | 1/3 | 6 | 3 |
| U12 | 1/5 | 1 | 1/6 | 2 | 1/3 |
| U13 | 3 | 6 | 1 | 6 | 4 |
| U14 | 1/6 | 1/2 | 1/6 | 1 | 1/3 |
| U15 | 1/3 | 3 | 1/4 | 3 | 1 |
| λmax = 5.2197 | CI = 0.0549 | CR = 0.0490 |  |  |  |

****Table D8. Pairwise comparison matrix — Expert 2 (Local value A1)****

|  | ****U11**** | ****U12**** | ****U13**** | ****U14**** | ****U15**** |
| --- | --- | --- | --- | --- | --- |
| U11 | 1 | 5 | 1/2 | 6 | 3 |
| U12 | 1/5 | 1 | 1/6 | 2 | 1/2 |
| U13 | 2 | 6 | 1 | 6 | 3 |
| U14 | 1/6 | 1/2 | 1/6 | 1 | 1/4 |
| U15 | 1/3 | 2 | 1/3 | 4 | 1 |
| λmax = 5.1195 | CI = 0.0299 | CR = 0.0267 |  |  |  |

****Table D9. Pairwise comparison matrix — Expert 3 (Local value A1)****

|  | ****U11**** | ****U12**** | ****U13**** | ****U14**** | ****U15**** |
| --- | --- | --- | --- | --- | --- |
| U11 | 1 | 1/5 | 4 | 1/6 | 1/2 |
| U12 | 5 | 1 | 5 | 1/4 | 2 |
| U13 | 1/4 | 1/5 | 1 | 1/6 | 1/3 |
| U14 | 6 | 4 | 6 | 1 | 3 |
| U15 | 2 | 1/2 | 3 | 1/3 | 1 |
| λmax = 5.3694 | CI = 0.0924 | CR = 0.0825 |  |  |  |

****Table D10. Pairwise comparison matrix — Expert 4 (Local value A1)****

|  | ****U11**** | ****U12**** | ****U13**** | ****U14**** | ****U15**** |
| --- | --- | --- | --- | --- | --- |
| U11 | 1 | 1/4 | 6 | 1/6 | 1/3 |
| U12 | 4 | 1 | 5 | 1/2 | 2 |
| U13 | 1/6 | 1/5 | 1 | 1/6 | 1/4 |
| U14 | 6 | 2 | 6 | 1 | 4 |
| U15 | 3 | 1/2 | 4 | 1/4 | 1 |
| λmax = 5.4245 | CI = 0.1061 | CR = 0.0948 |  |  |  |

****Table D11. Pairwise comparison matrix — Expert 5 (Local value A1)****

|  | ****U11**** | ****U12**** | ****U13**** | ****U14**** | ****U15**** |
| --- | --- | --- | --- | --- | --- |
| U11 | 1 | 3 | 1/6 | 5 | 2 |
| U12 | 1/3 | 1 | 1/6 | 2 | 1/2 |
| U13 | 6 | 6 | 1 | 6 | 4 |
| U14 | 1/5 | 1/2 | 1/6 | 1 | 1/3 |
| U15 | 1/2 | 2 | 1/4 | 3 | 1 |
| λmax = 5.2594 | CI = 0.0649 | CR = 0.0579 |  |  |  |

****2.2. Scientific value (A2) – four secondary indicators (U21 to U24)****

****Table D12. Pairwise comparison matrix — Expert 1 (Scientific value A2)****

|  | ****U21**** | ****U22**** | ****U23**** | ****U24**** |
| --- | --- | --- | --- | --- |
| U21 | 1 | 2 | 3 | 5 |
| U22 | 1/2 | 1 | 3 | 6 |
| U23 | 1/3 | 1/3 | 1 | 3 |
| U24 | 1/5 | 1/6 | 1/3 | 1 |
| λmax = 4.1109 | CI = 0.0370 | CR = 0.0415 |  |  |

****Table D13. Pairwise comparison matrix — Expert 2 (Scientific value A2)****

|  | ****U21**** | ****U22**** | ****U23**** | ****U24**** |
| --- | --- | --- | --- | --- |
| U21 | 1 | 3 | 4 | 6 |
| U22 | 1/3 | 1 | 2 | 5 |
| U23 | 1/4 | 1/2 | 1 | 6 |
| U24 | 1/6 | 1/5 | 1/6 | 1 |
| λmax = 4.2358 | CI = 0.0786 | CR = 0.0883 |  |  |

****Table D14. Pairwise comparison matrix — Expert 3 (Scientific value A2)****

|  | ****U21**** | ****U22**** | ****U23**** | ****U24**** |
| --- | --- | --- | --- | --- |
| U21 | 1 | 1/2 | 1/4 | 1/6 |
| U22 | 2 | 1 | 1/3 | 1/5 |
| U23 | 4 | 3 | 1 | 1/3 |
| U24 | 6 | 5 | 3 | 1 |
| λmax = 4.0787 | CI = 0.0262 | CR = 0.0295 |  |  |

****Table D15. Pairwise comparison matrix — Expert 4 (Scientific value A2)****

|  | ****U21**** | ****U22**** | ****U23**** | ****U24**** |
| --- | --- | --- | --- | --- |
| U21 | 1 | 1/2 | 1/3 | 1/4 |
| U22 | 2 | 1 | 1/2 | 1/4 |
| U23 | 3 | 2 | 1 | 1/6 |
| U24 | 4 | 4 | 6 | 1 |
| λmax = 4.2583 | CI = 0.0861 | CR = 0.0967 |  |  |

****Table D16. Pairwise comparison matrix — Expert 5 (Scientific value A2)****

|  | ****U21**** | ****U22**** | ****U23**** | ****U24**** |
| --- | --- | --- | --- | --- |
| U21 | 1 | 2 | 4 | 5 |
| U22 | 1/2 | 1 | 2 | 4 |
| U23 | 1/4 | 1/2 | 1 | 4 |
| U24 | 1/5 | 1/4 | 1/4 | 1 |
| λmax = 4.1301 | CI = 0.0434 | CR = 0.0487 |  |  |

****2.3. Historic value (A3) – six secondary indicators (U31 to U36)****

****Table D17. Pairwise comparison matrix — Expert 1 (Historic value A3)****

|  | ****U31**** | ****U32**** | ****U33**** | ****U34**** | ****U35**** | ****U36**** |
| --- | --- | --- | --- | --- | --- | --- |
| U31 | 1 | 1/3 | 1/6 | 4 | 1/4 | 3 |
| U32 | 3 | 1 | 1/6 | 5 | 1/3 | 4 |
| U33 | 6 | 6 | 1 | 6 | 2 | 5 |
| U34 | 1/4 | 1/5 | 1/6 | 1 | 1/5 | 1/2 |
| U35 | 4 | 3 | 1/2 | 5 | 1 | 5 |
| U36 | 1/3 | 1/4 | 1/5 | 2 | 1/5 | 1 |
| λmax = 6.5116 | CI = 0.1023 | CR = 0.0812 |  |  |  |  |

****Table D18. Pairwise comparison matrix — Expert 2 (Historic value A3)****

|  | ****U31**** | ****U32**** | ****U33**** | ****U34**** | ****U35**** | ****U36**** |
| --- | --- | --- | --- | --- | --- | --- |
| U31 | 1 | 1/2 | 1/4 | 5 | 1/3 | 2 |
| U32 | 2 | 1 | 1/6 | 6 | 1/2 | 4 |
| U33 | 4 | 6 | 1 | 6 | 2 | 5 |
| U34 | 1/5 | 1/6 | 1/6 | 1 | 1/5 | 1/2 |
| U35 | 3 | 2 | 1/2 | 5 | 1 | 5 |
| U36 | 1/2 | 1/4 | 1/5 | 2 | 1/5 | 1 |
| λmax = 6.3740 | CI = 0.0748 | CR = 0.0594 |  |  |  |  |

****Table D19. Pairwise comparison matrix — Expert 3 (Historic value A3)****

|  | ****U31**** | ****U32**** | ****U33**** | ****U34**** | ****U35**** | ****U36**** |
| --- | --- | --- | --- | --- | --- | --- |
| U31 | 1 | 3 | 5 | 1/3 | 4 | 1/2 |
| U32 | 1/3 | 1 | 6 | 1/6 | 5 | 1/3 |
| U33 | 1/5 | 1/6 | 1 | 1/6 | 1/2 | 1/5 |
| U34 | 3 | 6 | 6 | 1 | 5 | 2 |
| U35 | 1/4 | 1/5 | 2 | 1/5 | 1 | 1/5 |
| U36 | 2 | 3 | 5 | 1/2 | 5 | 1 |
| λmax = 6.5052 | CI = 0.1010 | CR = 0.0802 |  |  |  |  |

****Table D20. Pairwise comparison matrix — Expert 4 (Historic value A3)****

|  | ****U31**** | ****U32**** | ****U33**** | ****U34**** | ****U35**** | ****U36**** |
| --- | --- | --- | --- | --- | --- | --- |
| U31 | 1 | 3 | 5 | 1/5 | 4 | 1/4 |
| U32 | 1/3 | 1 | 5 | 1/4 | 3 | 1/3 |
| U33 | 1/5 | 1/5 | 1 | 1/6 | 1/2 | 1/5 |
| U34 | 5 | 4 | 6 | 1 | 5 | 2 |
| U35 | 1/4 | 1/3 | 2 | 1/5 | 1 | 1/5 |
| U36 | 4 | 3 | 5 | 1/2 | 5 | 1 |
| λmax = 6.5343 | CI = 0.1069 | CR = 0.0848 |  |  |  |  |

****Table D21. Pairwise comparison matrix — Expert 5 (Historic value A3)****

|  | ****U31**** | ****U32**** | ****U33**** | ****U34**** | ****U35**** | ****U36**** |
| --- | --- | --- | --- | --- | --- | --- |
| U31 | 1 | 1/2 | 1/5 | 6 | 1/4 | 3 |
| U32 | 2 | 1 | 1/5 | 6 | 1/4 | 4 |
| U33 | 5 | 5 | 1 | 6 | 2 | 5 |
| U34 | 1/6 | 1/6 | 1/6 | 1 | 1/5 | 1/2 |
| U35 | 4 | 4 | 1/2 | 5 | 1 | 5 |
| U36 | 1/3 | 1/4 | 1/5 | 2 | 1/5 | 1 |
| λmax = 6.5402 | CI = 0.1081 | CR = 0.0858 |  |  |  |  |

****2.4. Aesthetic value (A4) – five secondary indicators (U41 to U45)****

****Table D22. Pairwise comparison matrix — Expert 1 (Aesthetic value A4)****

|  | ****U41**** | ****U42**** | ****U43**** | ****U44**** | ****U45**** |
| --- | --- | --- | --- | --- | --- |
| U41 | 1 | 1/4 | 1/2 | 4 | 1/5 |
| U42 | 4 | 1 | 2 | 5 | 1/4 |
| U43 | 2 | 1/2 | 1 | 6 | 1/5 |
| U44 | 1/4 | 1/5 | 1/6 | 1 | 1/6 |
| U45 | 5 | 4 | 5 | 6 | 1 |
| λmax = 5.4019 | CI = 0.1005 | CR = 0.0897 |  |  |  |

****Table D23. Pairwise comparison matrix — Expert 2 (Aesthetic value A4)****

|  | ****U41**** | ****U42**** | ****U43**** | ****U44**** | ****U45**** |
| --- | --- | --- | --- | --- | --- |
| U41 | 1 | 1/4 | 1/3 | 2 | 1/6 |
| U42 | 4 | 1 | 2 | 4 | 1/4 |
| U43 | 3 | 1/2 | 1 | 3 | 1/5 |
| U44 | 1/2 | 1/4 | 1/3 | 1 | 1/6 |
| U45 | 6 | 4 | 5 | 6 | 1 |
| λmax = 5.2272 | CI = 0.0568 | CR = 0.0507 |  |  |  |

****Table D24. Pairwise comparison matrix — Expert 3 (Aesthetic value A4)****

|  | ****U41**** | ****U42**** | ****U43**** | ****U44**** | ****U45**** |
| --- | --- | --- | --- | --- | --- |
| U41 | 1 | 3 | 2 | 1/5 | 4 |
| U42 | 1/3 | 1 | 1/2 | 1/5 | 2 |
| U43 | 1/2 | 2 | 1 | 1/3 | 5 |
| U44 | 5 | 5 | 3 | 1 | 6 |
| U45 | 1/4 | 1/2 | 1/5 | 1/6 | 1 |
| λmax = 5.2619 | CI = 0.0655 | CR = 0.0585 |  |  |  |

****Table D25. Pairwise comparison matrix — Expert 4 (Aesthetic value A4)****

|  | ****U41**** | ****U42**** | ****U43**** | ****U44**** | ****U45**** |
| --- | --- | --- | --- | --- | --- |
| U41 | 1 | 3 | 2 | 1/2 | 6 |
| U42 | 1/3 | 1 | 1/2 | 1/6 | 3 |
| U43 | 1/2 | 2 | 1 | 1/4 | 6 |
| U44 | 2 | 6 | 4 | 1 | 6 |
| U45 | 1/6 | 1/3 | 1/6 | 1/6 | 1 |
| λmax = 5.1804 | CI = 0.0451 | CR = 0.0403 |  |  |  |

****Table D26. Pairwise comparison matrix — Expert 5 (Aesthetic value A4)****

|  | ****U41**** | ****U42**** | ****U43**** | ****U44**** | ****U45**** |
| --- | --- | --- | --- | --- | --- |
| U41 | 1 | 1/4 | 1/3 | 2 | 1/6 |
| U42 | 4 | 1 | 2 | 6 | 1/2 |
| U43 | 3 | 1/2 | 1 | 4 | 1/6 |
| U44 | 1/2 | 1/6 | 1/4 | 1 | 1/6 |
| U45 | 6 | 2 | 6 | 6 | 1 |
| λmax = 5.1973 | CI = 0.0493 | CR = 0.0440 |  |  |  |

****2.5. Sustainability value (A5) – four secondary indicators (U51 to U54)****

****Table D27. Pairwise comparison matrix — Expert 1 (Sustainability value A5)****

|  | ****U51**** | ****U52**** | ****U53**** | ****U54**** |
| --- | --- | --- | --- | --- |
| U51 | 1 | 1/4 | 1/6 | 1/3 |
| U52 | 4 | 1 | 1/3 | 3 |
| U53 | 6 | 3 | 1 | 4 |
| U54 | 3 | 1/3 | 1/4 | 1 |
| λmax = 4.1440 | CI = 0.0480 | CR = 0.0539 |  |  |

****Table D28. Pairwise comparison matrix — Expert 2 (Sustainability value A5)****

|  | ****U51**** | ****U52**** | ****U53**** | ****U54**** |
| --- | --- | --- | --- | --- |
| U51 | 1 | 1/4 | 1/6 | 1/2 |
| U52 | 4 | 1 | 1/2 | 4 |
| U53 | 6 | 2 | 1 | 5 |
| U54 | 2 | 1/4 | 1/5 | 1 |
| λmax = 4.0657 | CI = 0.0219 | CR = 0.0246 |  |  |

****Table D29. Pairwise comparison matrix — Expert 3 (Sustainability value A5)****

|  | ****U51**** | ****U52**** | ****U53**** | ****U54**** |
| --- | --- | --- | --- | --- |
| U51 | 1 | 3 | 6 | 2 |
| U52 | 1/3 | 1 | 6 | 1/2 |
| U53 | 1/6 | 1/6 | 1 | 1/3 |
| U54 | 1/2 | 2 | 3 | 1 |
| λmax = 4.2050 | CI = 0.0683 | CR = 0.0768 |  |  |

****Table D30. Pairwise comparison matrix — Expert 4 (Sustainability value A5)****

|  | ****U51**** | ****U52**** | ****U53**** | ****U54**** |
| --- | --- | --- | --- | --- |
| U51 | 1 | 3 | 5 | 2 |
| U52 | 1/3 | 1 | 5 | 1/2 |
| U53 | 1/5 | 1/5 | 1 | 1/5 |
| U54 | 1/2 | 2 | 5 | 1 |
| λmax = 4.1425 | CI = 0.0475 | CR = 0.0534 |  |  |

****Table D31. Pairwise comparison matrix — Expert 5 (Sustainability value A5)****

|  | ****U51**** | ****U52**** | ****U53**** | ****U54**** |
| --- | --- | --- | --- | --- |
| U51 | 1 | 1/3 | 1/4 | 1/2 |
| U52 | 3 | 1 | 1/2 | 2 |
| U53 | 4 | 2 | 1 | 3 |
| U54 | 2 | 1/2 | 1/3 | 1 |
| λmax = 4.0310 | CI = 0.0103 | CR = 0.0116 |  |  |

****Summary of Aggregated Group Consistency for Secondary Indicators:****

****Table D32. Aggregated Group Consistency for Secondary Indicators****

| **Indicator Group** | **λmax** | **CI** | **CR** |
| --- | --- | --- | --- |
| Local value (A1) | 5.0035 | 0.0009 | 0.0008 |
| Scientific value (A2) | 4.0056 | 0.0019 | 0.0021 |
| Historic value (A3) | 6.0229 | 0.0046 | 0.0036 |
| Aesthetic value (A4) | 5.0184 | 0.0046 | 0.0041 |
| Sustainability value (A5) | 4.0081 | 0.0027 | 0.0030 |

All CR values are below 0.1, indicating satisfactory consistency for all aggregated group judgment matrices. The resulting composite indicator weights are presented in Table 8 of the main manuscript.
