# Supplementary material for: An evaluation scale for the cultural value of heritage buildings
Source: PLoS One. 2026 Jun 23;21(6):e0350924. doi: 10.1371/journal.pone.0350924 (PMC13289924; doi:10.1371/journal.pone.0350924)
Supplement: Attachment — Submitted filename: Comments by the authors.docx [file pone.0350924.s005.docx]

**Comments by the authors**

**General evaluation**

Your study addresses an important problem and has potential value in Heritage Research and practice. The generic approach-which integrates qualitative insights with a systematic, multi-criteria methodology, and seeks to validate them-is promising. However, substantial revisions are required to ensure clarity, repeatability, and accuracy of the report in compliance with PLOS standards.

**1) Introduction**

- Clearly identify the research gap: explain the shortcomings of the current assessment frameworks, and the importance of this shortcoming.
- Add an explicit statement of contribution, explaining the new in your scale (its structure, dimensions, weighting method, validation methodology, or applicability).
- Be sure to back up all historical and Digital claims with appropriate references.
- Improve the academic structure so that the foreground is graded by:
- the background - problem - gap - goal / questions - contribution - research roadmap.

**2) Literature review**

- Reorganize the literature review into thematic subsections (e.g.: cultural value concepts; heritage assessment frameworks; indicator systems; participatory/experience-based models; scale development/validation).
- Go beyond the description: Add a critical comparison (differences in structures, indicators, methods, restrictions).
- Use the literature review to clearly justify your methodological choices (why this framework, why the hierarchical analysis method, why these indicators).

**3) Methodology (major clarity and reproducibility issues)**

Please provide sufficient detail so another researcher can replicate the study.

**3.1 Research design**

- - Identify the type of mixed curriculum design (sequential/concurrent/exploratory) and provide a flowchart linking each method to each research goal.

**3.2 Qualitative phase (interviews/FGDs)**

- - Report: number of participants / experts, inclusion criteria, experience profile, recruitment process, context.
- - Presented the structure of the interview protocol/focus group discussion (topics, number of questions, sample questions).

**3.3 Qualitative analysis (NVivo/coding)**

- Identify the analytical framework (e.g. objective analysis versus rooted theory), coding steps, iterations, and reliability checks (coders agreement or equivalent).
- Show traceability of raw text ---- icons ---- cursors (table/figure).

**3.4 Expert screening / indicator refinement**

- Specify whether the Delphi method or any other procedure is used; the number of rounds; decision rules; compatibility metrics.
- Provide a table showing the indicators before and after the examination, stating the reasons for deletion/merging

**3.5 AHP weighting**

- Show the hierarchy (goal - criteria - sub - criteria-indicators), the number of experts, the scale used, the grouping method, the number of arrays.
- Report consistency ratios (CR) and thresholds; include a table of final weights and rankings.

**3.6 Validation**

- Define reliability tests (e.g. cronbach's Alpha coefficient) and validity types (content/construction/convergence/discrimination).
- Report the sample size, standards/thresholds, provide tables with all verification outputs.

**4) Results (insufficient quantitative reporting)**

- Report exact numbers: indicators before/after screening, agreement levels, AHP weights and rankings, CR values, and all reliability/validity statistics.
- Add essential tables/figures: indicator hierarchy, screening outcomes, weight matrices/weight tables, validation tables, and relevant diagrams.
- Avoid generic statements (e.g., “satisfactory,” “significant”) without numerical evidence and/or tests.

**5) Discussion**

- - Associate each explanatory claim with specific results (weights, ratings, verification measures).
- - Compare the results with specific previous studies/frameworks (author/year) and indicate whether your results confirm or challenge them.
- - Make the effects practical: explain who uses the scale, when, and how it supports decision-making.
- - Clarify the scope and conditions of the circular based on your data and methodology.

**6) Conclusion**

- - Provide an evidence-based summary of the main quantitative findings.
- - Accurately reformulate the contribution and distinguish it from previous studies.
- - Clarify the limitations while clearly linking them to the methodologies and implications.
- - Provide specific future research directions derived from findings and limitations.

**7) References, tables, and figures**

- - Ensure that the list of references is complete and its formatting is consistent; check that all quotations contained in the text appear in the list and vice versa.
- - Addition of missing methodological references (mixed methodologies, analysis using the NVivo program/objective analysis, hierarchy analysis, scale validation).
- - Ensure that all figures / tables have independent explanatory headings and are mentioned in the text; clarify whether the figures are original or modified, citing sources accordingly.
