# Supplementary material for: An evaluation scale for the cultural value of heritage buildings
Source: PLoS One. 2026 Jun 23;21(6):e0350924. doi: 10.1371/journal.pone.0350924 (PMC13289924; doi:10.1371/journal.pone.0350924)
Supplement: Attachment — Submitted filename: Response to Reviewer 2.docx [file pone.0350924.s007.docx]

**Comments by the authors**

**General evaluation**

Your study addresses an important problem and has potential value in Heritage Research and practice. The generic approach-which integrates qualitative insights with a systematic, multi-criteria methodology, and seeks to validate them-is promising. However, substantial revisions are required to ensure clarity, repeatability, and accuracy of the report in compliance with PLOS standards.

**Response:**

Thank you for your professional, meticulous and patient guidance. This is the most detailed set of review comments I have received. I have benefited greatly from it and shall amend each point in accordance with your suggestions.

**1) Introduction**

- Clearly identify the research gap: explain the shortcomings of the current assessment frameworks, and the importance of this shortcoming.
- Add an explicit statement of contribution, explaining the new in your scale (its structure, dimensions, weighting method, validation methodology, or applicability).
- Be sure to back up all historical and Digital claims with appropriate references.
- Improve the academic structure so that the foreground is graded by:
- the background - problem - gap - goal / questions - contribution - research roadmap.

**Response:**

（1）The author has addressed the shortcomings of current assessment frameworks and their significance in the introduction, explicitly identifying research gaps.

（2）Contributions of this study have been supplemented by detailing the novel aspects of the proposed scale.

（3）Supporting references (References 16 and 24) for historical and numerical claimshave been added.

（4）Paragraph structure and article framework have been revised in response to reviewer comments.

**2) Literature review**

- Reorganize the literature review into thematic subsections (e.g.: cultural value concepts; heritage assessment frameworks; indicator systems; participatory/experience-based models; scale development/validation).
- Go beyond the description: Add a critical comparison (differences in structures, indicators, methods, restrictions).
- Use the literature review to clearly justify your methodological choices (why this framework, why the hierarchical analysis method, why these indicators).

**Response:**

（1）The author has separately drafted the Theoretical Basis section as per the guidance, treating it as an independent section. As the core objective of this article is to develop a cultural value evaluation scale without addressing specific value evaluations, and given that this section primarily references key conclusions from the author's previously accepted review article, it has been titled Theoretical Basis.

（2）The rationale for the methodological choices in this study will be elaborated in Section 3.1 (Research Design) of the Methodology section.

**3) Methodology (major clarity and reproducibility issues)**

Please provide sufficient detail so another researcher can replicate the study.

**3.1 Research design**

- - Identify the type of mixed curriculum design (sequential/concurrent/exploratory) and provide a flowchart linking each method to each research goal.

**Response:**
The flowchart has been optimised in Section 3.1, linking each method to its respective research objective.

**3.2 Qualitative phase (interviews/FGDs)**

- - Report: number of participants / experts, inclusion criteria, experience profile, recruitment process, context.
- - Presented the structure of the interview protocol/focus group discussion (topics, number of questions, sample questions).

**Response:**

（1）The interview report has been reviewed and refined in the methodology section (3.3.1, 3.3.2), with the interview question structure explained (Appendix A and B).

（2）The focus group discussion report has been reviewed and refined in the methodology section (3.4.1), with the focus group discussion question structure explained (Appendix C).

**3.3 Qualitative analysis (NVivo/coding)**

- Identify the analytical framework (e.g. objective analysis versus rooted theory), coding steps, iterations, and reliability checks (coders agreement or equivalent).
- Show traceability of raw text ---- icons ---- cursors (table/figure).

**Response:**

（1）The data analysis process (including analytical framework and coding procedures) has been elaborated in greater detail in sections 3.3.3 and 3.4.2.

（2）An iterative diagram of indicators (Figure 4) has been incorporated into section 4.3 to illustrate the traceability from raw text to icons to cursors.

（3）The authors have introduced a separate section on data reliability checks (5. Validation).

（4）As the findings from the interview method directly inform the focus group discussions, the authors have consolidated sections 3.2 and 3.3 into a single section. This section first presents a coherent account of the data collection and analysis process for the interview method, followed by a coherent account for the FGD (3.3 Data Collection and Analysis of Interviews, 3.4 Data Collection and Analysis of FGD).

**3.4 Expert screening / indicator refinement**

- Specify whether the Delphi method or any other procedure is used; the number of rounds; decision rules; compatibility metrics.
- Provide a table showing the indicators before and after the examination, stating the reasons for deletion/merging

**Response:**

（1）It has been explicitly stated in the article (Section 4.4. Clarification of Indicator Weights through AHP): ‘This section primarily outlines the weight calculation and consistency testing process of AHP, with the Delphi method primarily reflected in the construction phase of the judgement matrix.’

（2） Determining the scale content was one of the two phased tasks of the focus group discussions (the other being assigning weights to indicators), and the details of the data collection process were elaborated in the preceding section.

（3）The indicator iteration diagram（Fig 4） added in Section 4.3 illustrates changes across indicators before and after adjustments.

（4）The rationale for deleting/merging indicators is explained in the data analysis sections (3.3.3 and 3.4.2).

**3.5 AHP weighting**

- Show the hierarchy (goal - criteria - sub - criteria-indicators), the number of experts, the scale used, the grouping method, the number of arrays.
- Report consistency ratios (CR) and thresholds; include a table of final weights and rankings.

**Response:**

（1）Determining weights constitutes another task for experts participating in the focus group discussions. The authors have addressed this aspect in a separate section (4.4 Determination of Indicator Weights through AHP).

（2）Relevant content has been supplemented in accordance with the review comments. The final weights are presented in Table 8, with the ranking of each indicator reflected within the table and further clarified through Fig 6.

**3.6 Validation**

- Define reliability tests (e.g. cronbach's Alpha coefficient) and validity types (content/construction/convergence/discrimination).
- Report the sample size, standards/thresholds, provide tables with all verification outputs.

**Response:**

（1）Detailed information on validation has been supplemented (Section 5. Validation). This section tests the scale's reliability and validity through questionnaire surveys and provides tables with all verification outputs.

（2）As with the interview and FGD stages, researchers verbally assured respondents that personal information would be kept confidential during the questionnaire survey, obtaining consent before distributing the questionnaires (Section 5.1 Data collection during the testing phase).

**4) Results (insufficient quantitative reporting)**

- Report exact numbers: indicators before/after screening, agreement levels, AHP weights and rankings, CR values, and all reliability/validity statistics.
- Add essential tables/figures: indicator hierarchy, screening outcomes, weight matrices/weight tables, validation tables, and relevant diagrams.
- Avoid generic statements (e.g., “satisfactory,” “significant”) without numerical evidence and/or tests.

**Response:**

（1）The conclusions are primarily presented in section 4 (4. Generating a CVES for Heritage Buildings). The relevant data requested in the reviewer comments has been incorporated, and Fig 4 has been added to Section 4.3 to further elucidate the iterative process of the metrics.

（2）The article's generalised phrasing has been revised.

**5) Discussion**

- - Associate each explanatory claim with specific results (weights, ratings, verification measures).
- - Compare the results with specific previous studies/frameworks (author/year) and indicate whether your results confirm or challenge them.
- - Make the effects practical: explain who uses the scale, when, and how it supports decision-making.
- - Clarify the scope and conditions of the circular based on your data and methodology.

**Response:**

（1）Section 6 incorporates comparative analysis with the preceding framework, with particular emphasis on elucidating (and refining) the connotations of two newly introduced categories—Local Value and Sustainability Value—first proposed within this framework (6.1 The ‘Wide’ and ‘Narrow’ Concepts of Local Value, 6.2 The ‘New’ and ‘Old’ Concepts of Sustainability Value), while outlining their similarities and differences with corresponding categories in the prior framework.

（2）This study validates certain aspects of previous research while proposing refinement strategies based on critiques of some findings (supplemented in sections 6.1 and 6.2).

（3）Each explanatory claim is linked to specific outcomes (6.3. Differences in the weighting of indicators in the scale, 6.4. Similarities and Differences among Various Stakeholders).

（4）It clarifies who would use the scale, when it would be employed, and how it supports decision-making, thereby delineating the scope and conditions of this notification (6.5. Limitations and Future Research Directions).

**6) Conclusion**

- - Provide an evidence-based summary of the main quantitative findings.
- - Accurately reformulate the contribution and distinguish it from previous studies.
- - Clarify the limitations while clearly linking them to the methodologies and implications.
- - Provide specific future research directions derived from findings and limitations.

**Response:**
（1）The summary of results has been optimised.
（2）The key contributions of this article have been reformulated.

（3）The limitations and future research directions have been clarified.

**7) References, tables, and figures**

- - Ensure that the list of references is complete and its formatting is consistent; check that all quotations contained in the text appear in the list and vice versa.
- - Addition of missing methodological references (mixed methodologies, analysis using the NVivo program/objective analysis, hierarchy analysis, scale validation).
- - Ensure that all figures / tables have independent explanatory headings and are mentioned in the text; clarify whether the figures are original or modified, citing sources accordingly.

**Response:**

（1）The content and formatting of the references have been re-examined, with relevant literature added (References 16 and 24).

（2）The explanatory captions, annotations, and descriptions within the main text pertaining to the figures and tables have been reviewed and adjusted.
