# Supplementary material for: An evaluation scale for the cultural value of heritage buildings
Source: PLoS One. 2026 Jun 23;21(6):e0350924. doi: 10.1371/journal.pone.0350924 (PMC13289924; doi:10.1371/journal.pone.0350924)
Supplement: Attachment — Submitted filename: Response to Reviewers.docx [file pone.0350924.s008.docx]

Reviewer #1:

4. The manuscript is generally well written and logically structured. Minor language polishing could further improve clarity in some sections, but overall it is intelligible and meets standard academic English requirements.

**Response:**

We appreciate your affirmation. We have conducted a comprehensive review and optimized the language of some paragraphs (as can be seen in the "Revised Manuscript with Track Changes").

Editor:

First, the reliability check of the qualitative coding process (e.g., inter-coder agreement or a systematic verification procedure) is inadequately reported, making it difficult to assess the objectivity and reproducibility of the qualitative findings.

**Response:**

We thank the reviewer for this important comment. To address the reliability of the qualitative coding process, we have added a new inter-coder agreement procedure in Section 3.3.3. From the total of 20 interview transcripts (10 local shop owners + 10 visitors), 6 transcripts (30%) were randomly selected. A second independent researcher coded these transcripts, and Cohen‘s Kappa was calculated (κ = 0.84, 95% confidence interval: 0.78–0.90), indicating ‘almost perfect’ agreement according to Landis and Koch’s (1977) benchmark. Most disagreements occurred at the boundary between ‘local value’ and ‘historic value’ and were resolved through consensus meetings. This addition significantly improves the transparency and reproducibility of our qualitative findings.

Second, the transparency of how expert judgments were aggregated in AHP is lacking; the manuscript does not specify how the individual judgments of the five experts were combined into the group pairwise comparison matrix (e.g., whether the geometric mean was used, nor provides access to the raw individual matrices).

**Response:**

We agree that the aggregation process of expert judgments in AHP must be transparent. In the revised manuscript (Section 4.4.2), we have explicitly stated that the geometric mean was used to aggregate the five individual pairwise comparison matrices into a single group matrix. Furthermore, to ensure full reproducibility, we have now provided the raw individual judgment matrices from all five experts for both the primary and secondary indicators in a new Appendix E. This allows readers and reviewers to independently verify the weighting calculations.

**Additional Notes:**

In this revision, due to changes in the affiliation information of some authors, we have updated the relevant information accordingly and added a corresponding author. All authors of the paper have been informed and have given their consent to the above information.
