# Supplementary material for: An evaluation scale for the cultural value of heritage buildings
Source: PLoS One. 2026 Jun 23;21(6):e0350924. doi: 10.1371/journal.pone.0350924 (PMC13289924; doi:10.1371/journal.pone.0350924)
Supplement: Peer review history

# Peer review: Decision Letter 0

**DOI:** 10.1371/journal.pone.0350924.r001

**Authors:** Baohua Wen

2 Jan 2026

PONE-D-25-59893

An Evaluation Scale for the Cultural Value of Heritage Buildings

PLOS One

Dear Dr. Huang,

Thank you for submitting your manuscript to PLOS ONE. After careful consideration, we feel that it has merit but does not fully meet PLOS ONE’s publication criteria as it currently stands. Therefore, we invite you to submit a revised version of the manuscript that addresses the points raised during the review process.

==============================

**The current version does not meet the publication criteria due to overall weaknesses in:**

(1) the theoretical framework (lack of clarity of the research gap and contribution),

(2) methodological transparency and reproducibility (not precisely defined procedures at all stages), and

(3) Evidence-Based Reporting (presenting the results narratively with a lack of quantitative details). In particular, the manuscript lacks basic information regarding expert/sample selection, decision-making rules, hierarchical analysis consistency reports, and reliability/validity statistics. The tables / figures required for transparency and interpretability are also absent or insufficient. For more detailed feedback, please refer to the reviewers' comments.

==============================

Please submit your revised manuscript by Feb 16 2026 11:59PM. If you will need more time than this to complete your revisions, please reply to this message or contact the journal office at plosone@plos.org. When you're ready to submit your revision, log on to https://www.editorialmanager.com/pone/ and select the 'Submissions Needing Revision' folder to locate your manuscript file.

Please include the following items when submitting your revised manuscript:

- A letter that responds to each point raised by the academic editor and reviewer(s). You should upload this letter as a separate file labeled 'Response to Reviewers'.
- A marked-up copy of your manuscript that highlights changes made to the original version. You should upload this as a separate file labeled 'Revised Manuscript with Track Changes'.
- An unmarked version of your revised paper without tracked changes. You should upload this as a separate file labeled 'Manuscript'.

If you would like to make changes to your financial disclosure, please include your updated statement in your cover letter. Guidelines for resubmitting your figure files are available below the reviewer comments at the end of this letter.

If applicable, we recommend that you deposit your laboratory protocols in protocols.io to enhance the reproducibility of your results. Protocols.io assigns your protocol its own identifier (DOI) so that it can be cited independently in the future. For instructions see: https://journals.plos.org/plosone/s/submission-guidelines#loc-laboratory-protocols. Additionally, PLOS ONE offers an option for publishing peer-reviewed Lab Protocol articles, which describe protocols hosted on protocols.io. Read more information on sharing protocols at https://plos.org/protocols?utm_medium=editorial-email&utm_source=authorletters&utm_campaign=protocols.

We look forward to receiving your revised manuscript.

Kind regards,

Baohua Wen

Academic Editor

PLOS One

**Journal Requirements:**

1. When submitting your revision, we need you to address these additional requirements.

Please ensure that your manuscript meets PLOS ONE's style requirements, including those for file naming. The PLOS ONE style templates can be found at

https://journals.plos.org/plosone/s/file?id=wjVg/PLOSOne_formatting_sample_main_body.pdf and

https://journals.plos.org/plosone/s/file?id=ba62/PLOSOne_formatting_sample_title_authors_affiliations.pdf

2. Please provide additional details regarding participant consent. In the ethics statement in the Methods and online submission information, please ensure that you have specified (a) whether consent was informed and (b) what type you obtained (for instance, written or verbal, and if verbal, how it was documented and witnessed). If your study included minors, state whether you obtained consent from parents or guardians. If the need for consent was waived by the ethics committee, please include this information.

If you are reporting a retrospective study of medical records or archived samples, please ensure that you have discussed whether all data were fully anonymized before you accessed them and/or whether the IRB or ethics committee waived the requirement for informed consent. If patients provided informed written consent to have data from their medical records used in research, please include this information.

3. We note that Figures 2 and 3 in your submission contain copyrighted images. All PLOS content is published under the Creative Commons Attribution License (CC BY 4.0), which means that the manuscript, images, and Supporting Information files will be freely available online, and any third party is permitted to access, download, copy, distribute, and use these materials in any way, even commercially, with proper attribution. For more information, see our copyright guidelines: http://journals.plos.org/plosone/s/licenses-and-copyright.

We require you to either (a) present written permission from the copyright holder to publish these figures specifically under the CC BY 4.0 license, or (b) remove the figures from your submission:

a. You may seek permission from the original copyright holder of Figures 2 and 3 to publish the content specifically under the CC BY 4.0 license.

We recommend that you contact the original copyright holder with the Content Permission Form (http://journals.plos.org/plosone/s/file?id=7c09/content-permission-form.pdf) and the following text:

“I request permission for the open-access journal PLOS ONE to publish XXX under the Creative Commons Attribution License (CCAL) CC BY 4.0 (http://creativecommons.org/licenses/by/4.0/). Please be aware that this license allows unrestricted use and distribution, even commercially, by third parties. Please reply and provide explicit written permission to publish XXX under a CC BY license and complete the attached form.”

Please upload the completed Content Permission Form or other proof of granted permissions as an "Other" file with your submission.

In the figure caption of the copyrighted figure, please include the following text: “Reprinted from [ref] under a CC BY license, with permission from [name of publisher], original copyright [original copyright year].”

b. If you are unable to obtain permission from the original copyright holder to publish these figures under the CC BY 4.0 license or if the copyright holder’s requirements are incompatible with the CC BY 4.0 license, please either i) remove the figure or ii) supply a replacement figure that complies with the CC BY 4.0 license. Please check copyright information on all replacement figures and update the figure caption with source information. If applicable, please specify in the figure caption text when a figure is similar but not identical to the original image and is therefore for illustrative purposes only.

4. We note that this data set consists of interview transcripts. Can you please confirm that all participants gave consent for interview transcript to be published?

If they DID provide consent for these transcripts to be published, please also confirm that the transcripts do not contain any potentially identifying information (or let us know if the participants consented to having their personal details published and made publicly available). We consider the following details to be identifying information:

- Names, nicknames, and initials

- Age more specific than round numbers

- GPS coordinates, physical addresses, IP addresses, email addresses

- Information in small sample sizes (e.g. 40 students from X class in X year at X university)

- Specific dates (e.g. visit dates, interview dates)

- ID numbers

Or, if the participants DID NOT provide consent for these transcripts to be published:

- Provide a de-identified version of the data or excerpts of interview responses

- Provide information regarding how these transcripts can be accessed by researchers who meet the criteria for access to confidential data, including:

a) the grounds for restriction

b) the name of the ethics committee, Institutional Review Board, or third-party organization that is imposing sharing restrictions on the data

c) a non-author, institutional point of contact that is able to field data access queries, in the interest of maintaining long-term data accessibility.

d) Any relevant data set names, URLs, DOIs, etc. that an independent researcher would need in order to request your minimal data set.

For further information on sharing data that contains sensitive participant information, please see: https://journals.plos.org/plosone/s/data-availability#loc-human-research-participant-data-and-other-sensitive-data

If there are ethical, legal, or third-party restrictions upon your dataset, you must provide all of the following details (https://journals.plos.org/plosone/s/data-availability#loc-acceptable-data-access-restrictions):

a) A complete description of the dataset

b) The nature of the restrictions upon the data (ethical, legal, or owned by a third party) and the reasoning behind them

c) The full name of the body imposing the restrictions upon your dataset (ethics committee, institution, data access committee, etc)

d) If the data are owned by a third party, confirmation of whether the authors received any special privileges in accessing the data that other researchers would not have

e) Direct, non-author contact information (preferably email) for the body imposing the restrictions upon the data, to which data access requests can be sent

5. We note that there is identifying data in the Supporting Information file “Fig S1”. Due to the inclusion of these potentially identifying data, we have removed this file from your file inventory. Prior to sharing human research participant data, authors should consult with an ethics committee to ensure data are shared in accordance with participant consent and all applicable local laws.

Data sharing should never compromise participant privacy. It is therefore not appropriate to publicly share personally identifiable data on human research participants. The following are examples of data that should not be shared:

-Name, initials, physical address

-Ages more specific than whole numbers

-Internet protocol (IP) address

-Specific dates (birth dates, death dates, examination dates, etc.)

-Contact information such as phone number or email address

-Location data

-ID numbers that seem specific (long numbers, include initials, titled “Hospital ID”) rather than random (small numbers in numerical order)

Data that are not directly identifying may also be inappropriate to share, as in combination they can become identifying. For example, data collected from a small group of participants, vulnerable populations, or private groups should not be shared if they involve indirect identifiers (such as sex, ethnicity, location, etc.) that may risk the identification of study participants.

Additional guidance on preparing raw data for publication can be found in our Data Policy (https://journals.plos.org/plosone/s/data-availability#loc-human-research-participant-data-and-other-sensitive-data) and in the following article: http://www.bmj.com/content/340/bmj.c181.long.

Please remove or anonymize all personal information (<specific identifying information in file to be removed>), ensure that the data shared are in accordance with participant consent, and re-upload a fully anonymized data set. Please note that spreadsheet columns with personal information must be removed and not hidden as all hidden columns will appear in the published file.

6. We notice that your supplementary figures are uploaded with the file type 'Figure'. Please amend the file type to 'Supporting Information'. Please ensure that each Supporting Information file has a legend listed in the manuscript after the references list.

7. If the reviewer comments include a recommendation to cite specific previously published works, please review and evaluate these publications to determine whether they are relevant and should be cited. There is no requirement to cite these works unless the editor has indicated otherwise.

[Note: HTML markup is below. Please do not edit.]

Reviewers' comments:

Reviewer's Responses to Questions

**Comments to the Author**

1. Is the manuscript technically sound, and do the data support the conclusions?

The manuscript must describe a technically sound piece of scientific research with data that supports the conclusions. Experiments must have been conducted rigorously, with appropriate controls, replication, and sample sizes. The conclusions must be drawn appropriately based on the data presented.

Reviewer #1: Yes

Reviewer #2: Partly

**********

2. Has the statistical analysis been performed appropriately and rigorously?

Reviewer #1: Yes

Reviewer #2: I Don't Know

**********

3. Have the authors made all data underlying the findings in their manuscript fully available?

The PLOS Data policy requires authors to make all data underlying the findings described in their manuscript fully available without restriction, with rare exception (please refer to the Data Availability Statement in the manuscript PDF file). The data should be provided as part of the manuscript or its supporting information, or deposited to a public repository. For example, in addition to summary statistics, the data points behind means, medians and variance measures should be available. If there are restrictions on publicly sharing data—e.g. participant privacy or use of data from a third party—those must be specified.

Reviewer #1: Yes

Reviewer #2: No

**********

4. Is the manuscript presented in an intelligible fashion and written in standard English?

PLOS ONE does not copyedit accepted manuscripts, so the language in submitted articles must be clear, correct, and unambiguous. Any typographical or grammatical errors should be corrected at revision, so please note any specific errors here.

Reviewer #1: Yes

Reviewer #2: Yes

**********

5. Review Comments to the Author

Please use the space provided to explain your answers to the questions above. You may also include additional comments for the author, including concerns about dual publication, research ethics, or publication ethics. (Please upload your review as an attachment if it exceeds 20,000 characters)

**Reviewer #1:**  1.The manuscript addresses an important gap in heritage studies by proposing a structured evaluation scale for the cultural value of heritage buildings. The overall research design is coherent, and the mixed-methods approach (systematic review, interviews, FGD, and AHP) is appropriate for the stated objectives. The procedures for data collection and analysis are generally well described, and the conclusions are supported by the presented qualitative and quantitative results.

2.However, there are several issues that should be addressed before publication. First, the empirical basis is limited to a single case study, which restricts the generalizability of the proposed scale; this limitation should be stated more explicitly. Second, although AHP is applied, the justification for the number of experts and the robustness of the weighting process could be discussed more critically. Third, the manuscript would benefit from clearer reporting on how qualitative findings were translated into specific indicators and items, to improve transparency and reproducibility. Finally, the English language is generally understandable but contains repetitive phrasing, awkward constructions, and minor grammatical errors that should be corrected to improve clarity.

3.Overall, the study is technically sound and potentially suitable for publication in PLOS ONE after minor to moderate revision.

**Reviewer #2:**  Comments by the authors

General evaluation

Your study addresses an important problem and has potential value in Heritage Research and practice. The generic approach-which integrates qualitative insights with a systematic, multi-criteria methodology, and seeks to validate them-is promising. However, substantial revisions are required to ensure clarity, repeatability, and accuracy of the report in compliance with PLOS standards.

1) Introduction

- Clearly identify the research gap: explain the shortcomings of the current assessment frameworks, and the importance of this shortcoming.

- Add an explicit statement of contribution, explaining the new in your scale (its structure, dimensions, weighting method, validation methodology, or applicability).

- Be sure to back up all historical and Digital claims with appropriate references.

- Improve the academic structure so that the foreground is graded by:

- the background - problem - gap - goal / questions - contribution - research roadmap.

2) Literature review

- Reorganize the literature review into thematic subsections (e.g.: cultural value concepts; heritage assessment frameworks; indicator systems; participatory/experience-based models; scale development/validation).

- Go beyond the description: Add a critical comparison (differences in structures, indicators, methods, restrictions).

- Use the literature review to clearly justify your methodological choices (why this framework, why the hierarchical analysis method, why these indicators).

3) Methodology (major clarity and reproducibility issues)

Please provide sufficient detail so another researcher can replicate the study.

3.1 Research design

- - Identify the type of mixed curriculum design (sequential/concurrent/exploratory) and provide a flowchart linking each method to each research goal.

3.2 Qualitative phase (interviews/FGDs)

- - Report: number of participants / experts, inclusion criteria, experience profile, recruitment process, context.

- - Presented the structure of the interview protocol/focus group discussion (topics, number of questions, sample questions).

3.3 Qualitative analysis (NVivo/coding)

- Identify the analytical framework (e.g. objective analysis versus rooted theory), coding steps, iterations, and reliability checks (coders agreement or equivalent).

- Show traceability of raw text ---- icons ---- cursors (table/figure).

3.4 Expert screening / indicator refinement

- Specify whether the Delphi method or any other procedure is used; the number of rounds; decision rules; compatibility metrics.

- Provide a table showing the indicators before and after the examination, stating the reasons for deletion/merging

3.5 AHP weighting

- Show the hierarchy (goal - criteria - sub - criteria-indicators), the number of experts, the scale used, the grouping method, the number of arrays.

- Report consistency ratios (CR) and thresholds; include a table of final weights and rankings.

3.6 Validation

- Define reliability tests (e.g. cronbach's Alpha coefficient) and validity types (content/construction/convergence/discrimination).

- Report the sample size, standards/thresholds, provide tables with all verification outputs.

4) Results (insufficient quantitative reporting)

- Report exact numbers: indicators before/after screening, agreement levels, AHP weights and rankings, CR values, and all reliability/validity statistics.

- Add essential tables/figures: indicator hierarchy, screening outcomes, weight matrices/weight tables, validation tables, and relevant diagrams.

- Avoid generic statements (e.g., “satisfactory,” “significant”) without numerical evidence and/or tests.

5) Discussion

- - Associate each explanatory claim with specific results (weights, ratings, verification measures).

- - Compare the results with specific previous studies/frameworks (author/year) and indicate whether your results confirm or challenge them.

- - Make the effects practical: explain who uses the scale, when, and how it supports decision-making.

- - Clarify the scope and conditions of the circular based on your data and methodology.

6) Conclusion

- - Provide an evidence-based summary of the main quantitative findings.

- - Accurately reformulate the contribution and distinguish it from previous studies.

- - Clarify the limitations while clearly linking them to the methodologies and implications.

- - Provide specific future research directions derived from findings and limitations.

7) References, tables, and figures

- - Ensure that the list of references is complete and its formatting is consistent; check that all quotations contained in the text appear in the list and vice versa.

- - Addition of missing methodological references (mixed methodologies, analysis using the NVivo program/objective analysis, hierarchy analysis, scale validation).

- - Ensure that all figures / tables have independent explanatory headings and are mentioned in the text; clarify whether the figures are original or modified, citing sources accordingly.

**********

6. PLOS authors have the option to publish the peer review history of their article (what does this mean?). If published, this will include your full peer review and any attached files.

If you choose “no”, your identity will remain anonymous but your review may still be made public.

**Do you want your identity to be public for this peer review?**   For information about this choice, including consent withdrawal, please see our Privacy Policy.

Reviewer #1: No

Reviewer #2: No

**********

[NOTE: If reviewer comments were submitted as an attachment file, they will be attached to this email and accessible via the submission site. Please log into your account, locate the manuscript record, and check for the action link "View Attachments". If this link does not appear, there are no attachment files.]

To ensure your figures meet our technical requirements, please review our figure guidelines: https://journals.plos.org/plosone/s/figures

You may also use PLOS’s free figure tool, NAAS, to help you prepare publication quality figures: https://journals.plos.org/plosone/s/figures#loc-tools-for-figure-preparation.

NAAS will assess whether your figures meet our technical requirements by comparing each figure against our figure specifications.

Attachment Submitted filename: Comments by the authors.docx

# Author comment: Author response to Decision Letter 1

**DOI:** 10.1371/journal.pone.0350924.r002

6 Feb 2026

Response to reviewer 1:

Thank you for your kind words. I shall continue to strive diligently (Please see Attachment “Response to reviewer 1” for a detailed response).

Response to reviewer 1:

Thank you for your professional, meticulous and patient guidance. This is the most detailed set of review comments I have received. I have benefited greatly from it and shall amend each point in accordance with your suggestions (Please see Attachment “Response to reviewer 2” for a detailed response).

Attachment Submitted filename: Response to Reviewer 2.docx

# Peer review: Decision Letter 1

**DOI:** 10.1371/journal.pone.0350924.r003

**Authors:** Baohua Wen

8 Apr 2026

PONE-D-25-59893R1

An Evaluation Scale for the Cultural Value of Heritage Buildings

PLOS One

Dear Dr. Huang,

Thank you for submitting your manuscript to PLOS ONE. After careful consideration, we feel that it has merit but does not fully meet PLOS ONE’s publication criteria as it currently stands. Therefore, we invite you to submit a revised version of the manuscript that addresses the points raised during the review process.

================================

I concur with Reviewer 2 that the authors have made a conscientious and substantially improved response to the reviewers’ comments, and have incorporated many beneficial revisions into the manuscript. Nevertheless, after careful re-evaluation, two core methodological issues remain insufficiently addressed. First, the reliability check of the qualitative coding process (e.g., inter-coder agreement or a systematic verification procedure) is inadequately reported, making it difficult to assess the objectivity and reproducibility of the qualitative findings. Second, the transparency of how expert judgments were aggregated in AHP is lacking; the manuscript does not specify how the individual judgments of the five experts were combined into the group pairwise comparison matrix (e.g., whether the geometric mean was used, nor provides access to the raw individual matrices). These issues are essential for ensuring the methodological rigor and credibility of the conclusions. Therefore, the authors are strongly encouraged to address these two points in detail and provide supporting documentation in the next revision.

================================

Please submit your revised manuscript by May 23 2026 11:59PM. If you will need more time than this to complete your revisions, please reply to this message or contact the journal office at plosone@plos.org. When you're ready to submit your revision, log on to https://www.editorialmanager.com/pone/ and select the 'Submissions Needing Revision' folder to locate your manuscript file.

Please include the following items when submitting your revised manuscript:

- A letter that responds to each point raised by the academic editor and reviewer(s). You should upload this letter as a separate file labeled 'Response to Reviewers'.
- A marked-up copy of your manuscript that highlights changes made to the original version. You should upload this as a separate file labeled 'Revised Manuscript with Track Changes'.
- An unmarked version of your revised paper without tracked changes. You should upload this as a separate file labeled 'Manuscript'.

If you would like to make changes to your financial disclosure, please include your updated statement in your cover letter. Guidelines for resubmitting your figure files are available below the reviewer comments at the end of this letter.

If applicable, we recommend that you deposit your laboratory protocols in protocols.io to enhance the reproducibility of your results. Protocols.io assigns your protocol its own identifier (DOI) so that it can be cited independently in the future. For instructions see: https://journals.plos.org/plosone/s/submission-guidelines#loc-laboratory-protocols. Additionally, PLOS ONE offers an option for publishing peer-reviewed Lab Protocol articles, which describe protocols hosted on protocols.io. Read more information on sharing protocols at https://plos.org/protocols?utm_medium=editorial-email&utm_source=authorletters&utm_campaign=protocols.

As the corresponding author, your ORCID iD is verified in the submission system and will appear in the published article. PLOS supports the use of ORCID, and we encourage all coauthors to register for an ORCID iD and use it as well. Please encourage your coauthors to verify their ORCID iD within the submission system before final acceptance, as unverified ORCID iDs will not appear in the published article. *Only*   the individual author can complete the verification step; PLOS staff *cannot*   verify ORCID iDs on behalf of authors.

We look forward to receiving your revised manuscript.

Kind regards,

Baohua Wen

Academic Editor

PLOS One

Journal Requirements:

If the reviewer comments include a recommendation to cite specific previously published works, please review and evaluate these publications to determine whether they are relevant and should be cited. There is no requirement to cite these works unless the editor has indicated otherwise.

[Note: HTML markup is below. Please do not edit.]

Reviewers' comments:

Reviewer's Responses to Questions

**Comments to the Author**

1. If the authors have adequately addressed your comments raised in a previous round of review and you feel that this manuscript is now acceptable for publication, you may indicate that here to bypass the “Comments to the Author” section, enter your conflict of interest statement in the “Confidential to Editor” section, and submit your "Accept" recommendation.

Reviewer #1: All comments have been addressed

Reviewer #2: (No Response)

**********

2. Is the manuscript technically sound, and do the data support the conclusions?

The manuscript must describe a technically sound piece of scientific research with data that supports the conclusions. Experiments must have been conducted rigorously, with appropriate controls, replication, and sample sizes. The conclusions must be drawn appropriately based on the data presented.

Reviewer #1: Yes

Reviewer #2: (No Response)

**********

3. Has the statistical analysis been performed appropriately and rigorously?

Reviewer #1: Yes

Reviewer #2: (No Response)

**********

4. Have the authors made all data underlying the findings in their manuscript fully available?

The PLOS Data policy requires authors to make all data underlying the findings described in their manuscript fully available without restriction, with rare exception (please refer to the Data Availability Statement in the manuscript PDF file). The data should be provided as part of the manuscript or its supporting information, or deposited to a public repository. For example, in addition to summary statistics, the data points behind means, medians and variance measures should be available. If there are restrictions on publicly sharing data—e.g. participant privacy or use of data from a third party—those must be specified.

Reviewer #1: Yes

Reviewer #2: (No Response)

**********

5. Is the manuscript presented in an intelligible fashion and written in standard English?

PLOS ONE does not copyedit accepted manuscripts, so the language in submitted articles must be clear, correct, and unambiguous. Any typographical or grammatical errors should be corrected at revision, so please note any specific errors here.

Reviewer #1: Yes

Reviewer #2: (No Response)

**********

6. Review Comments to the Author

Please use the space provided to explain your answers to the questions above. You may also include additional comments for the author, including concerns about dual publication, research ethics, or publication ethics. (Please upload your review as an attachment if it exceeds 20,000 characters)

Reviewer #1: 1.The manuscript presents a clear and systematic approach to developing a Cultural Value Evaluation Scale (CVES) for heritage buildings. The integration of multiple methods, including literature review, face-to-face interviews, go-along interviews, and focus group discussions (FGD), strengthens the robustness of the research design. The use of NVivo for qualitative analysis and AHP for weighting indicators is appropriate and well justified.

2.The study successfully incorporates perspectives from different stakeholders, including local shop owners, visitors, and experts, which enhances the comprehensiveness and applicability of the proposed framework. The development of the LHSSA framework (Local, Historic, Sustainability, Scientific, and Aesthetic values) is a meaningful contribution to the field.

3.The data collection process appears rigorous, with clear procedures for achieving saturation in interviews and systematic coding and validation processes. The addition of the sustainability dimension improves the relevance of the scale in contemporary heritage conservation discourse.

4.The manuscript is generally well written and logically structured. Minor language polishing could further improve clarity in some sections, but overall it is intelligible and meets standard academic English requirements.

5.No major concerns regarding research ethics or data availability were identified. The authors have clearly stated that all data are available within the manuscript and supporting files, and ethical considerations such as informed consent and anonymity were appropriately addressed.

Reviewer #2: (No Response)

**********

7. PLOS authors have the option to publish the peer review history of their article (what does this mean?). If published, this will include your full peer review and any attached files.

If you choose “no”, your identity will remain anonymous but your review may still be made public.

**Do you want your identity to be public for this peer review?**   For information about this choice, including consent withdrawal, please see our Privacy Policy.

Reviewer #1: **Yes:**  Adib Amany

Reviewer #2: **Yes:**  Al Fahmawee, Emad Al Dein Hasan

**********

[NOTE: If reviewer comments were submitted as an attachment file, they will be attached to this email and accessible via the submission site. Please log into your account, locate the manuscript record, and check for the action link "View Attachments". If this link does not appear, there are no attachment files.]

To ensure your figures meet our technical requirements, please review our figure guidelines: https://journals.plos.org/plosone/s/figures

You may also use PLOS’s free figure tool, NAAS, to help you prepare publication quality figures: https://journals.plos.org/plosone/s/figures#loc-tools-for-figure-preparation.

NAAS will assess whether your figures meet our technical requirements by comparing each figure against our figure specifications.

# Author comment: Author response to Decision Letter 2

**DOI:** 10.1371/journal.pone.0350924.r004

6 May 2026

Reviewer #1:

4.The manuscript is generally well written and logically structured. Minor language polishing could further improve clarity in some sections, but overall it is intelligible and meets standard academic English requirements.

Response:

We appreciate your affirmation. We have conducted a comprehensive review and optimized the language of some paragraphs (as can be seen in the "Revised Manuscript with Track Changes").

Editor:

First, the reliability check of the qualitative coding process (e.g., inter-coder agreement or a systematic verification procedure) is inadequately reported, making it difficult to assess the objectivity and reproducibility of the qualitative findings.

Response:

We thank the reviewer for this important comment. To address the reliability of the qualitative coding process, we have added a new inter-coder agreement procedure in Section 3.3.3. From the total of 20 interview transcripts (10 local shop owners + 10 visitors), 6 transcripts (30%) were randomly selected. A second independent researcher coded these transcripts, and Cohen‘s Kappa was calculated (κ = 0.84, 95% confidence interval: 0.78–0.90), indicating ‘almost perfect’ agreement according to Landis and Koch’s (1977) benchmark. Most disagreements occurred at the boundary between ‘local value’ and ‘historic value’ and were resolved through consensus meetings. This addition significantly improves the transparency and reproducibility of our qualitative findings.

Second, the transparency of how expert judgments were aggregated in AHP is lacking; the manuscript does not specify how the individual judgments of the five experts were combined into the group pairwise comparison matrix (e.g., whether the geometric mean was used, nor provides access to the raw individual matrices).

Response:

We agree that the aggregation process of expert judgments in AHP must be transparent. In the revised manuscript (Section 4.4.2), we have explicitly stated that the geometric mean was used to aggregate the five individual pairwise comparison matrices into a single group matrix. Furthermore, to ensure full reproducibility, we have now provided the raw individual judgment matrices from all five experts for both the primary and secondary indicators in a new Appendix E. This allows readers and reviewers to independently verify the weighting calculations.

Attachment Submitted filename: Response to Reviewers.docx

# Peer review: Decision Letter 2

**DOI:** 10.1371/journal.pone.0350924.r005

**Authors:** Baohua Wen

20 May 2026

An Evaluation Scale for the Cultural Value of Heritage Buildings

PONE-D-25-59893R2

Dear Dr. Huang,

We’re pleased to inform you that your manuscript has been judged scientifically suitable for publication and will be formally accepted for publication once it meets all outstanding technical requirements.

Within one week, you’ll receive an e-mail detailing the required amendments. When these have been addressed, you’ll receive a formal acceptance letter and your manuscript will be scheduled for publication.

An invoice will be generated when your article is formally accepted. Please note, if your institution has a publishing partnership with PLOS and your article meets the relevant criteria, all or part of your publication costs will be covered. Please make sure your user information is up-to-date by logging into Editorial Manager at Editorial Manager® and clicking the ‘Update My Information' link at the top of the page. For questions related to billing, please contact billing support.

If your institution or institutions have a press office, please notify them about your upcoming paper to help maximize its impact. If they’ll be preparing press materials, please inform our press team as soon as possible -- no later than 48 hours after receiving the formal acceptance. Your manuscript will remain under strict press embargo until 2 pm Eastern Time on the date of publication. For more information, please contact onepress@plos.org.

Kind regards,

Baohua Wen

Academic Editor

PLOS One

Additional Editor Comments (optional):

Reviewers' comments:

Reviewer's Responses to Questions

**Comments to the Author**

1. If the authors have adequately addressed your comments raised in a previous round of review and you feel that this manuscript is now acceptable for publication, you may indicate that here to bypass the “Comments to the Author” section, enter your conflict of interest statement in the “Confidential to Editor” section, and submit your "Accept" recommendation.

Reviewer #1: All comments have been addressed

Reviewer #2: All comments have been addressed

**********

2. Is the manuscript technically sound, and do the data support the conclusions?

The manuscript must describe a technically sound piece of scientific research with data that supports the conclusions. Experiments must have been conducted rigorously, with appropriate controls, replication, and sample sizes. The conclusions must be drawn appropriately based on the data presented.

Reviewer #1: Yes

Reviewer #2: Yes

**********

3. Has the statistical analysis been performed appropriately and rigorously?

Reviewer #1: Yes

Reviewer #2: Yes

**********

4. Have the authors made all data underlying the findings in their manuscript fully available?

The PLOS Data policy requires authors to make all data underlying the findings described in their manuscript fully available without restriction, with rare exception (please refer to the Data Availability Statement in the manuscript PDF file). The data should be provided as part of the manuscript or its supporting information, or deposited to a public repository. For example, in addition to summary statistics, the data points behind means, medians and variance measures should be available. If there are restrictions on publicly sharing data—e.g. participant privacy or use of data from a third party—those must be specified.

Reviewer #1: Yes

Reviewer #2: Yes

**********

5. Is the manuscript presented in an intelligible fashion and written in standard English?

PLOS ONE does not copyedit accepted manuscripts, so the language in submitted articles must be clear, correct, and unambiguous. Any typographical or grammatical errors should be corrected at revision, so please note any specific errors here.

Reviewer #1: Yes

Reviewer #2: Yes

**********

6. Review Comments to the Author

Please use the space provided to explain your answers to the questions above. You may also include additional comments for the author, including concerns about dual publication, research ethics, or publication ethics. (Please upload your review as an attachment if it exceeds 20,000 characters)

Reviewer #1: In this second revision (R2), the authors have successfully addressed the previous methodological ambiguities. The qualitative phase now includes a robust inter-coder reliability check, yielding a Cohen's Kappa of κ = 0.84, which indicates an excellent level of agreement. Furthermore, the aggregation of expert judgments within the AHP has been clarified as utilizing the geometric mean, and the transparency criteria are fully satisfied by providing the raw pairwise comparison matrices in Appendix E.

The quantitative validation phase is statistically sound and rigorously performed. The internal consistency of the multi-dimensional scale is verified using Cronbach’s alpha, with all key value dimensions exceeding the 0.80 threshold (ranging from 0.871 to 0.941). Construct and convergence validity are appropriately tested using Principal Component Analysis (PCA) with a satisfactory cumulative explained variance of 77.68% (KMO = 0.745, Bartlett's test p = 0.000).

The authors have complied fully with the PLOS ONE Data Availability Policy. All minimal datasets, derived indicator scores, and calculation metrics are embedded within the manuscript and its supporting information files, including the newly added Appendix E for raw AHP matrices.

The phrasing and overall narrative structure have been heavily polished across the revisions. The manuscript is well-organized, intelligible, and adheres to standard international academic English publishing guidelines.

Reviewer #2: The revised manuscript reflects a clear attempt to address the concerns raised previously. In particular, improvements in methodological transparency, clarification of the qualitative coding process, reporting of inter-coder reliability procedures, and clarification of the AHP process have strengthened the manuscript.

**********

7. PLOS authors have the option to publish the peer review history of their article (what does this mean?). If published, this will include your full peer review and any attached files.

If you choose “no”, your identity will remain anonymous but your review may still be made public.

**Do you want your identity to be public for this peer review?**   For information about this choice, including consent withdrawal, please see our Privacy Policy.

Reviewer #1: **Yes:**  Adib Amany

Reviewer #2: No

**********

# Editor report: Acceptance letter

**DOI:** 10.1371/journal.pone.0350924.r006

**Authors:** Baohua Wen

PONE-D-25-59893R2

PLOS One

Dear Dr. Huang,

I'm pleased to inform you that your manuscript has been deemed suitable for publication in PLOS One. Congratulations! Your manuscript is now being handed over to our production team.

At this stage, our production department will prepare your paper for publication. This includes ensuring the following:

* All references, tables, and figures are properly cited

* All relevant supporting information is included in the manuscript submission,

* There are no issues that prevent the paper from being properly typeset

You will receive further instructions from the production team, including instructions on how to review your proof when it is ready. Please keep in mind that we are working through a large volume of accepted articles, so please give us a few days to review your paper and let you know the next and final steps.

Lastly, if your institution or institutions have a press office, please let them know about your upcoming paper now to help maximize its impact. If they'll be preparing press materials, please inform our press team within the next 48 hours. Your manuscript will remain under strict press embargo until 2 pm Eastern Time on the date of publication. For more information, please contact onepress@plos.org.

You will receive an invoice from PLOS for your publication fee after your manuscript has reached the completed accept phase. If you receive an email requesting payment before acceptance or for any other service, this may be a phishing scheme. Learn how to identify phishing emails and protect your accounts at https://explore.plos.org/phishing.

If we can help with anything else, please email us at customercare@plos.org.

Thank you for submitting your work to PLOS ONE and supporting open access.

Kind regards,

PLOS ONE Editorial Office Staff

on behalf of

Dr. Baohua Wen

Academic Editor

PLOS One
